# Modeling the impact of the Omicron infection wave in Germany

**DOI:** 10.1093/biomethods/bpad005

**Published:** 2023-03-21

**Authors:** Benjamin F Maier, Angelique Burdinski, Marc Wiedermann, Annika H Rose, Frank Schlosser, Matthias an der Heiden, Ole Wichmann, Thomas Harder, Dirk Brockmann

**Affiliations:** Robert Koch Institute, Berlin 13353, Germany; DTU Compute, Technical University of Denmark, Kongens Lyngby 2800, Denmark; Copenhagen Center for Social Data Science, University of Copenhagen, Copenhagen 1353, Denmark; Robert Koch Institute, Berlin 13353, Germany; Institute for Theoretical Biology and Integrated Research Institute for the Life-Sciences, Humboldt University of Berlin, Berlin 10115, Germany; Robert Koch Institute, Berlin 13353, Germany; Institute for Theoretical Biology and Integrated Research Institute for the Life-Sciences, Humboldt University of Berlin, Berlin 10115, Germany; Robert Koch Institute, Berlin 13353, Germany; Institute for Theoretical Biology and Integrated Research Institute for the Life-Sciences, Humboldt University of Berlin, Berlin 10115, Germany; Robert Koch Institute, Berlin 13353, Germany; Institute for Theoretical Biology and Integrated Research Institute for the Life-Sciences, Humboldt University of Berlin, Berlin 10115, Germany; Robert Koch Institute, Berlin 13353, Germany; Robert Koch Institute, Berlin 13353, Germany; Robert Koch Institute, Berlin 13353, Germany; Robert Koch Institute, Berlin 13353, Germany; Institute for Theoretical Biology and Integrated Research Institute for the Life-Sciences, Humboldt University of Berlin, Berlin 10115, Germany

**Keywords:** COVID-19, SARS-CoV-2, Omicron, infectious-disease modeling, forecast

## Abstract

In November 2021, the first infection with severe acute respiratory syndrome coronavirus 2 (SARS-CoV-2) variant of concern (VOC) B.1.1.529 (‘Omicron’) was reported in Germany, alongside global reports of reduced vaccine efficacy (VE) against infections with this variant. The potential threat posed by its rapid spread in Germany was, at the time, difficult to predict. We developed a variant-dependent population-averaged susceptible-exposed-infected-recovered infectious-disease model that included information about variant-specific and waning VEs based on empirical data available at the time. Compared to other approaches, our method aimed for minimal structural and computational complexity and therefore enabled us to respond to changes in the situation in a more agile manner while still being able to analyze the potential influence of (non-)pharmaceutical interventions (NPIs) on the emerging crisis. Thus, the model allowed us to estimate potential courses of upcoming infection waves in Germany, focusing on the corresponding burden on intensive care units (ICUs), the efficacy of contact reduction strategies, and the success of the booster vaccine rollout campaign. We expected a large cumulative number of infections with the VOC Omicron in Germany with ICU occupancy likely remaining below capacity, nevertheless, even without additional NPIs. The projected figures were in line with the actual Omicron waves that were subsequently observed in Germany with respective peaks occurring in mid-February and mid-March. Most surprisingly, our model showed that early, strict, and short contact reductions could have led to a strong ‘rebound’ effect with high incidences after the end of the respective NPIs, despite a potentially successful booster campaign. The results presented here informed legislation in Germany. The methodology developed in this study might be used to estimate the impact of future waves of COVID-19 or other infectious diseases.

## Introduction

During the COVID-19 pandemic, infectious-disease modeling proved to be an essential tool to estimate the impact of upcoming waves of the disease under different scenario assumptions regarding pathogen properties as well as pharmaceutical and nonpharmaceutical interventions (NPIs) [1–3]. Knowledge about the order of magnitude of an upcoming crisis can help to inform legislation regarding interventions to prevent public health systems and critical infrastructure from overburdening and collapsing [3–5]. With continuously emerging SARS-CoV-2 variants of concern (VOC) that can have relatively heterogeneous properties like virulence and severity of disease, model-based analyses continue to provide valuable insights into upcoming challenges and potential mitigation strategies [6].

With the impact of its upcoming dispersion being unclear, prior to the VOC B.1.1.529 (‘Omicron’) becoming the dominant strain of SARS-CoV-2 in Germany, we modeled possible trajectories of the upcoming infection wave in 2022, taking into account a variety of parameter estimates calibrated on the growth behavior of Omicron cases and cases of the VOC B.1.617.2 (‘Delta’) still prevalent in December 2021. Despite sustained high vaccine effectiveness against severe courses of the disease [7], reduced efficacy against infection was suspected to lead to higher growth rates and large outbreaks and, therefore, a potentially high burden on the healthcare system and critical infrastructure [8]. In order to estimate this potential impact, the spread of Omicron could have in Germany, and how potential NPI and pharmaceutical (i.e. vaccination) intervention might affect the trajectory of its spread, we devised and analyzed a parsimonious infectious-disease model in this study. Our objective was, at the time, to reduce model complexity as much as possible in order to draw resilient conclusions based on a limited amount of data in a short amount of time. As such, our analysis was based on the (partially limited) available empirical data regarding the properties of the VOCs Omicron and Delta as well as vaccine efficacies (VEs) and expectations of the booster distribution campaign, with an additional focus on the expected variation of contact behavior. As data on both variant’s mean latent and infectious period were, at the time, hard to obtain, we iterated our analyses for combinations of plausible assumptions, which allow for simple interpolation of modeling results in case better estimates of these parameters are found later on. Here, we publish and reflect on our methodology and results that were originally made public as a technical report in German on 3 February 2022, see [9].

**Figure 1:**
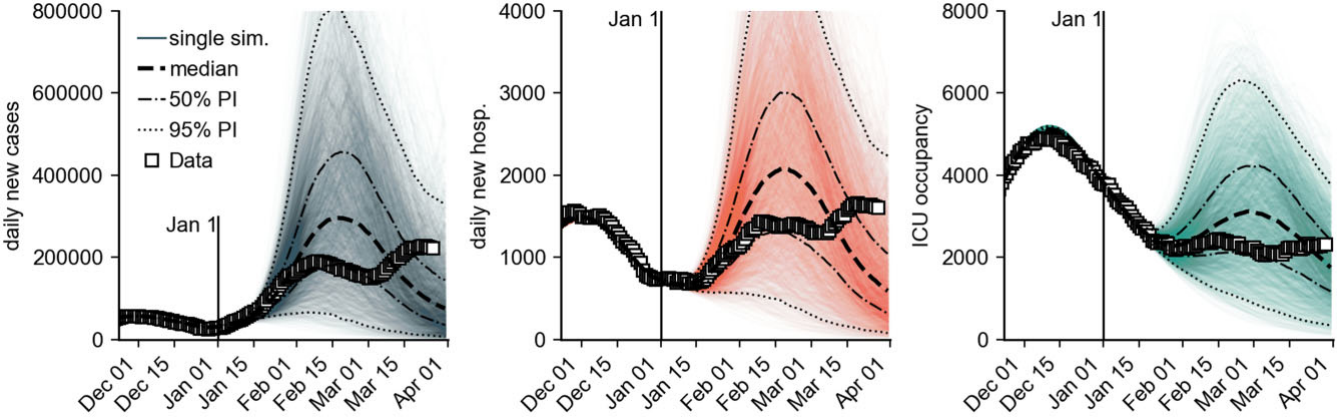
Model simulation results for the number of new cases per day, new hospitalizations per day and ICU occupancy for a combination of different model assumptions (see Materials and methods). Iterated here were ‘medium reach’ of the booster campaign (80% of those initially immunized receive a booster vaccination), ‘low VE’ and ‘high VE’ of the booster (VE, see Materials and methods section), different generation times [5 days, 4 days (Omicron latency: 2 days), 4 days (Omicron latency: 2 days), 4 days (Omicron latency: 1 day), and 3 days), as well as ‘no further contact reduction’ and ‘–20% contact reduction during the period from Jan 31 to Mar 15’. We defined the latency period as the mean duration between infection and onset of infectiousness. For each scenario combination, 180 simulations were performed, each with an individual stochastic contact modulation curve (see Materials and methods section). Shown are (i) individual simulation results (colored opaque lines), (ii) the median across all model runs (black dashed line) with 50% and 95% PIs, and (iii) the observed data (square data points). The RR of hospitalization by Omicron versus Delta was assumed to be *RR *=* *0.35 and intensive care *RR *=* *0.15. The model was calibrated until 1 January 2022, so simulations differ from that date onward.

Our model analysis predicted, at the time, a maximum median incidence of approximately 300 000 [50% prediction interval (PI) in 1000: 181–454, 95% PI in 1000: 55–804) reported cases per day with the median peak occurring in the mid of February 2022, reaching a cumulative Omicron case count of 16.5 million (50% PI in mio: 11.4–21.3, 95% PI in mio: 4.1–27.9) until 1 April 2022, see Table 1. Here, a 50% PI refers to the interquartile range of the predicted distribution of values (analogously for the 95% PI). These figures were in line with the actual Omicron waves that were subsequently observed in Germany (cf. Fig. 1) with respective peaks occurring in mid-February (peak: 191k daily new cases) and mid-March (peak: 230k daily new cases), cumulatively infecting a reported 14.8 million individuals during the study period. We found that the model peak height strongly depended on variations in the assumed generation time and decreased with a shorter generation time, which highlights the importance of reliable empirical estimates of this epidemiological parameter that were difficult to come by at the time. Regarding the efficacy of contact-reducing NPIs, low contact reductions were expected to lead to containment, whereas early, strict, and short contact reductions could have had an adverse effect after the respective lifting of restrictions at a later time, due to a waning of population-averaged VE during restrictions. Based on our results, we estimated that the relative risk (RR) of requiring intensive care for an infection with Omicron compared to an infection with Delta must assume values of the order of 10–20% to prevent the recurrence of extreme intensive care unit (ICU) burden, which was later found to be the case [10–12]. A hypothetically higher number of first-time immunizations (in our example, 15 million additional first-time immunizations) would have, in turn, greatly reduced the risk of large waves and maximally burdened ICUs, as well.

**Table 1:** Model prediction of the peak height of the different observables (median and PIs at the date of maximum median), see also Fig. 1

Quantity	Scenario median [in 1000]	50% PI	95% PI	Observed
New cases per day	296.2	180.9–453.6	55.1–803.6	230.1
New hospitalizations per day	2.1	1.3–3.0	0.5–4.9	1.7
ICU occupancy	3.1	2.1–4.2	0.9–6.3	2.5

Here, PIs refer to the respective inter-percentile ranges of the predicted distributions, for instance, the interquartile range in case of the 50% PI. In case of reported new cases per day and new hospitalizations per day, the reported observed values refer to the maximum of the 7-day running averages of the respective time series. In the nonaveraged time series, single greater values have been observed (e.g. a maximum of 247.8k reported new cases in the nonaveraged time series).

Our results informed legislation in Germany, and are, retrospectively, in good agreement with the actual course of the pandemic in Germany during the first quarter of 2022. We argue that the simple, yet effective, methodology used herein will be useful to estimate the impact of forthcoming waves of COVID-19 or other infectious diseases.

## Materials and methods

### Concise summary of methodology

In the following, we briefly outline our methodology to facilitate reader comprehension—a detailed description is provided in the following subsections.

**Figure 2:**
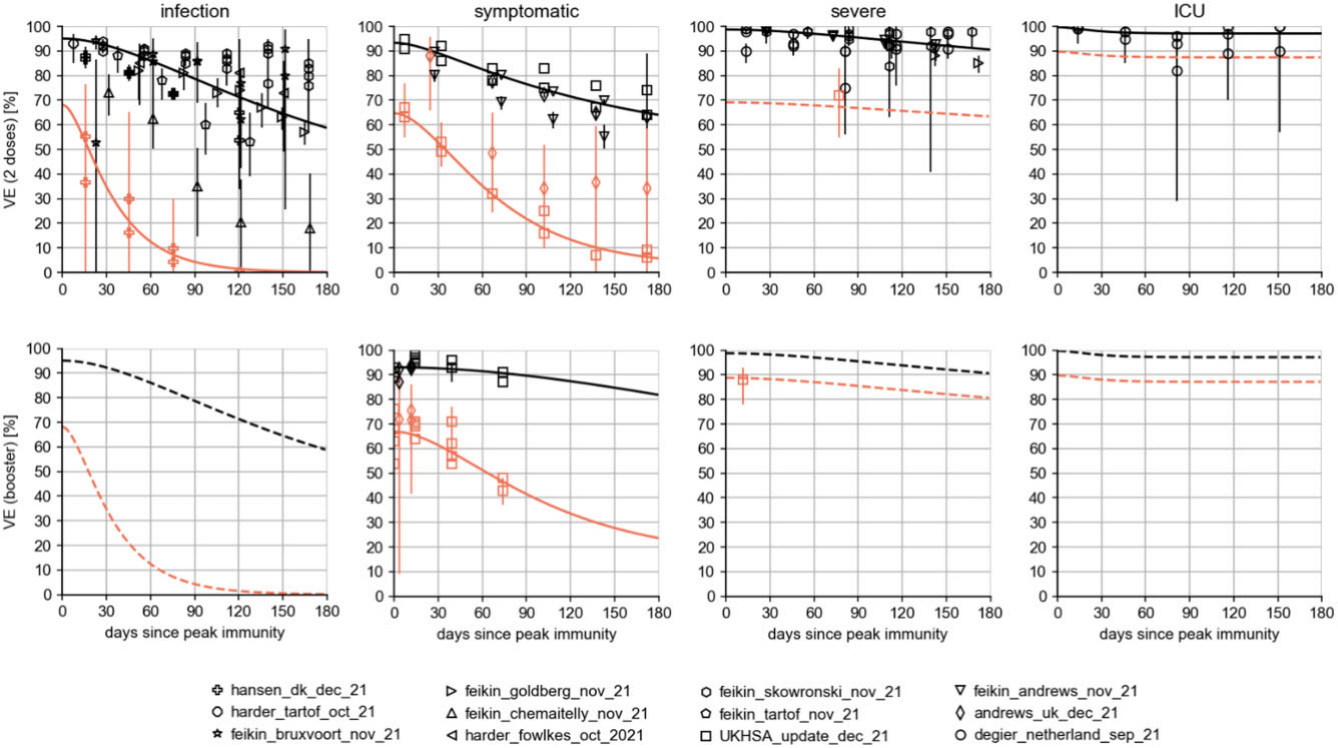
VEs for Omicron (orange) and Delta (black) following immunization. The top row shows the efficacies after basic immunization and the bottom row (where data were available) shows the corresponding values after booster immunization. Solid lines are the results of numerical fits of Equation (33); dashed lines indicate assumptions made. Data were compiled from a total of 12 studies (see list below the figure and ‘Population-wide vaccine efficacies’ section). For the ‘low VE’ scenario, it was assumed that the respective VEs against *infection* after the booster dose were equal to the effect of the vaccines after receiving two doses (pessimistic). For the ‘high VE’ scenario, we instead assumed that any VEs against infection were functionally equal to the time courses of VEs against *symptomatic disease* (optimistic).

**Figure 3:**
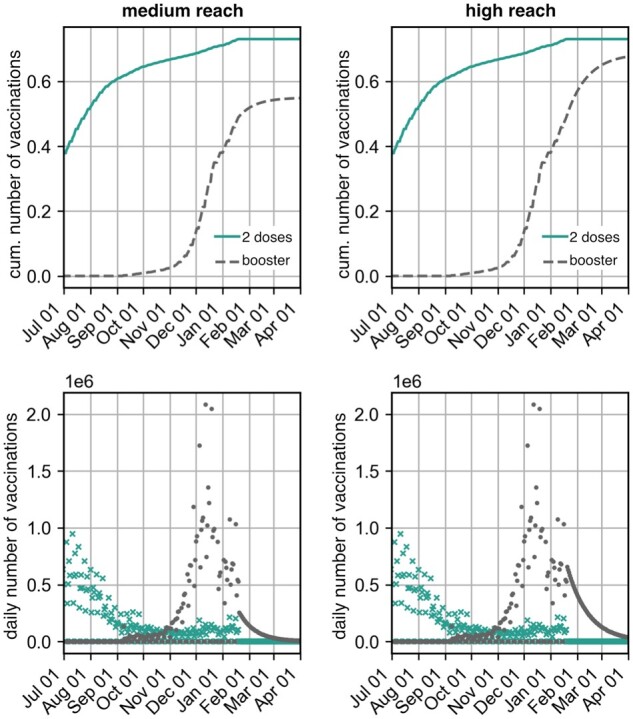
Cumulative number of vaccinated persons and number of daily vaccinations (data and extrapolation) assuming that the number of booster vaccinations reached 80% (medium reach) or 100% (high reach) of those first vaccinated by the end of 2021.

**Figure 4:**
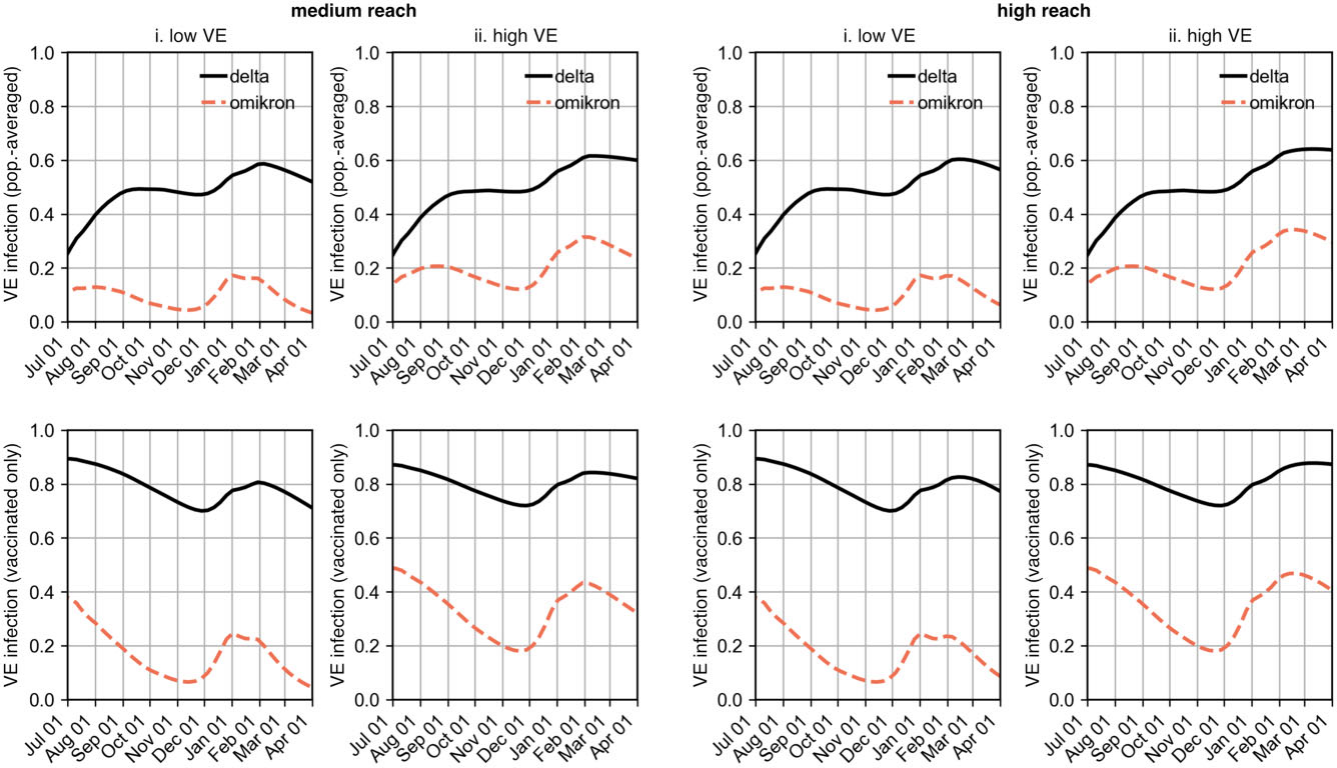
Population-wide VE $s_{v}(t)$ (top row, Equation (26)) and VE of the vaccinated population ${\tilde{s}}_{v}(t)$ (bottom row, Equation (27)) against infection under the assumed vaccination efficacies from Fig. 2. (i) Results according to the data for VE against infection of first-immunized (low VE) and (ii) assuming that VE against infection matched the data for VE against symptomatic infection (high VE). Both scenarios (low VE and high VE) were each considered under the assumption that either 80% (medium reach) or 100% (high reach) of those first immunized by the end of 2021 received booster vaccination. Note that using Farrington’s method, the ‘medium reach, high VE’ scenario combination accurately reflected the observed time series retrospectively, cf. Supplementary Analysis 1.1 and Supplementary Fig. S2.

**Figure 5:**
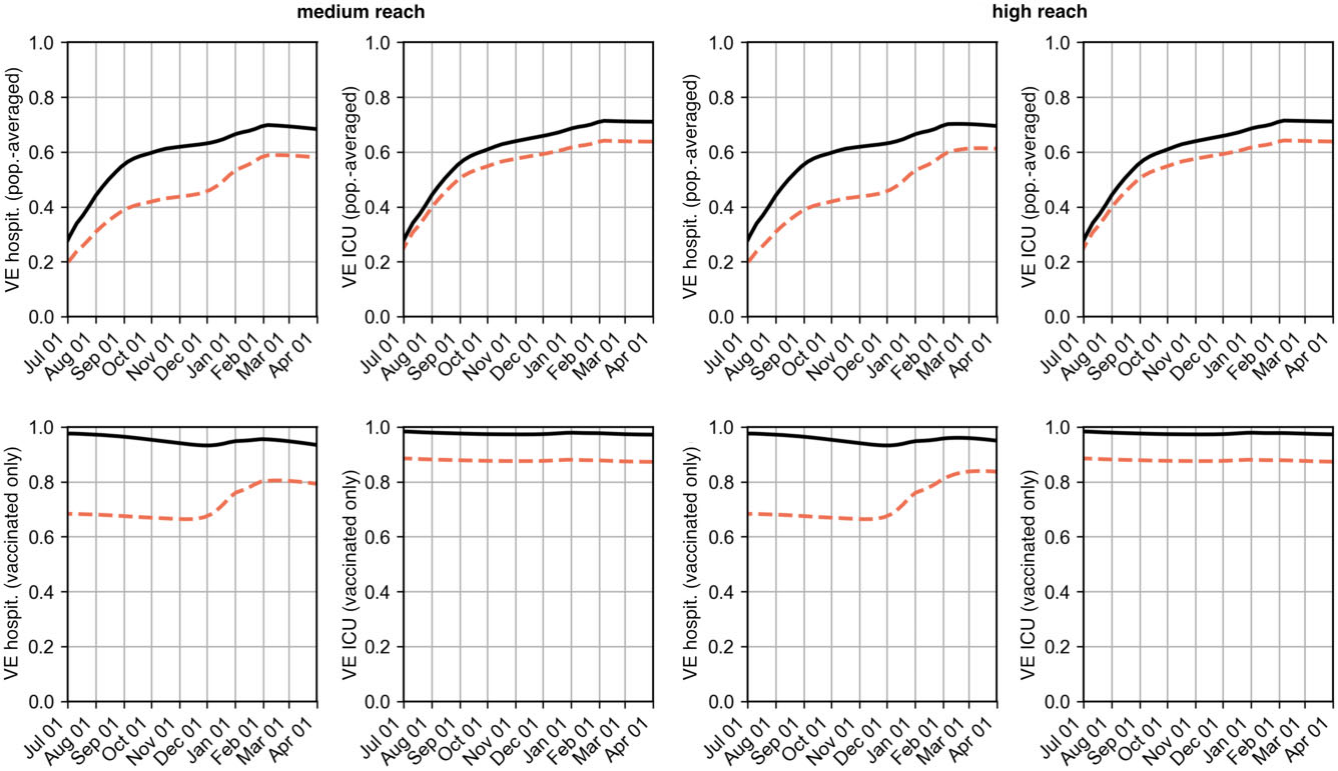
Population-wide VEs (top row, Equation (26)) and VEs of the vaccinated population (bottom row, Equation (27)) against hospitalization $h_{v}(t)$ and ${\tilde{h}}_{v}(t)$ as well as intensive care $u_{v}(t)$ and ${\tilde{u}}_{v}(t)$ under the assumed VEs from Fig. 2. Both scenarios (low VE and high VE) were each considered under the assumption that either 80% (medium reach) or 100% (high reach) of those first immunized by the end of 2021 receive booster vaccination. Solid lines show VE against Delta and dashed lines show VE against Omicron.

The model followed a population-averaged susceptible–exposed–infected–recovered (SEIR) dynamic, in which no explicit distinction was made between vaccinated and unvaccinated individuals or age strata [see ‘Model definition’ section, Equations (1–4)]. Instead, the impact of vaccines was modeled using population-averaged time-dependent VEs that were informed by vaccine attenuation curves and assumptions regarding the success of the booster campaign (see ‘Population-wide vaccine efficacies’ section and Figs 2–5). Mathematically, this approach yields the same growth rate as models that explicitly differentiate between vaccinated and unvaccinated persons, but, in doing so, it systematically overestimates the magnitude of large outbreaks by ∼10% in the worst case (see Supplementary Section 1.1 and Supplementary Fig. S1). The advantage of this simplified approach was that the model could be quickly adjusted and analyzed, as well as approximated analytically, allowing for an adaptive response to changes in the data.

Estimated and assumed time courses of VEs are shown in Fig. 2, see also ‘Population-wide vaccine efficacies’ section. Since mRNA vaccines account for the absolute majority of vaccine doses in Germany, only VEs of BioNTech and Moderna vaccines were considered in the following. We initially assumed that immune evasion of the Omicron variant corresponded to preliminary data from Denmark (hereinafter referred to as ‘low VE’, low vaccine efficacy) [13]. For a secondary analysis, we instead chose time courses of efficacy against infection that were functionally similar to time courses of efficacy against symptomatic disease, mostly observed in the UK, see [7]. This corresponded to a scenario with lower immune evasion and a stronger effect of booster vaccination, that is an optimistic scenario (hereafter referred to as ‘high VE’).

We further assumed that the booster vaccination campaign would have reached (i) 80% of the individuals (referred to as ‘medium reach’) or (ii) all individuals who received full vaccination protection in 2021 (referred to as ‘high reach’), see Fig. 3. The number of newly completed vaccination series (‘2 doses’) was ignored for the main analyses, but, for an illustrative analysis, the percentage was momentarily raised to ∼90% (by artificially increasing the number of initially vaccinated persons by 15 million) to show a hypothetical course of a scenario with a high initial immunization rate.

We varied the mean latency [2 days for Delta, as well as (i) 2 days for Omicron and (ii) 1 day for Omicron] and mean infectious period (upper bound: 3 days, lower bound: 2 days), that is simulated scenarios for generation times of 5, 4, and 3 days, see ‘Latency and infectious period’ section. We defined the latency period as the mean duration between infection and the onset of infectiousness.

The model was calibrated up to 1 January 2022 (contact modulation and VEs). To this end, the temporal contact modulation was inferred by mapping the model incidence to the observed incidence for the respective assumptions, see ‘Calibration of the contact modulation’ section. To emulate contact behavior similar to the observed one, stochastic simulations were performed to generate contact modulation curves that had the same statistical properties as the contact modulation observed in December 2021, see ‘Extrapolation of the contact modulation’ section. A vanishing variance was assumed for mean curves. Calibration of the model using the reported incidence of Delta cases showed increasing uncertainty as the proportion of Omicron cases grew, thus rendering the calibration increasingly unstable after 1 January 2022. After this date, only the daily vaccination data up to 22 January were updated and used in the results presented.

High reported infection rates can lead to behavioral changes and contact reductions [14]. For illustrative purposes, we assumed a contact reduction of –20% relative to the original courses, as well as higher contact reductions for complementary analyses, see ‘Contact reduction’ section.

In the UK and the USA, low values of RR of severe courses from infections with Omicron versus Delta were observed [10, 11, 15]. Thus, for each scenario, we determined the range of maximal possible RRs requiring intensive care in order to keep the ICU burden at most at the level of the burden in the previous wave (‘Delta wave’), see Supplementary Tables S1 and S2. For the analyses presented here, we assumed values of RR of hospitalization to be RR=0.35 and intensive care RR=0.15 to approximate the observed trajectories of hospitalization incidence and ICU occupancy observed in early January 2022.

The results discussed here are with regard to the originally spreading Omicron sublineage (BA.1), ignoring the influence of the sublineage BA.2, which did not spread substantially before early 2022.

### Model definition

Infection dynamics follow a temporally forced SEIR model:


(1) $$\partial_{t}S=-f(t)S\sum_{v}\;[1-s_{v}(t)]\;\alpha_{v}I_{v}$$

(2) $$\partial_{t}E_{v}=f(t)[1-s_{v}(t)]S\alpha_{v}I_{v}-\omega_{v}E_{v}$$

(3) $$\partial_{t}I_{v}=\omega_{v}E_{v}-\beta_{v}I_{v}$$

(4) $$\partial_{t}R_{v}=\beta_{v}I_{v}.$$

Here, *v* is a ‘variant of concern’ (hereinafter ‘variant’ or ‘VOC’), *f*(*t*) controls for time-varying contact behavior (e.g. through NPIs or voluntary behavioral change), $s_{v}(t)$ is the population-averaged efficacy of vaccination against infection (averaged across unvaccinated and vaccinated subpopulations). The transmissibility *α*, mean latency 1/ω, and mean infectious period 1/β are potentially variant dependent.

To fit the model to the data, we add compartments for (i) reported, (ii) hospitalized, or (iii) in need of intensive care. Let $p_{C,v}$ denotes the probability of appearing in the reporting statistics after infection (compartments *C*), $p_{H,v}$ the probability of an unvaccinated person being hospitalized after infection (i.e. becoming ‘severely’ ill with COVID-19, compartments *H*), and $p_{U,v}$ the likelihood of an unvaccinated individual requiring intensive care following infection (compartments *U*). Furthermore, $h_{v}(t)$ and $u_{v}(t)$ are time-dependent functions that quantify population-wide VEs against hospitalization and intensive care.

To adequately reflect the properties of the respective empirical waiting time distributions (for instance, an initial onset period), we split transitions between the susceptible compartment and reporting, hospitalization, and ICU admission into chains of exponential distributions, leading to overall waiting times that follow Erlang distributions with number *n* and rate n/τ (i.e. distributions with mean *τ* and standard deviation $\tau /\sqrt{n}$) to the transition times between infection and reporting, hospitalization, and ICU admission.

Thus, we obtain


(5) $$\partial_{t}C_{v}^{0}=p_{C,v}f(t)[1-s_{v}(t)]S\alpha_{v}I_{v}-\frac{n_{C,v}}{\tau_{C,v}}C_{v}^{0}$$

(6) $$\partial_{t}C_{v}^{i}=\frac{n_{C,v}}{\tau_{C,v}}C_{v}^{i-1}-\frac{n_{C,v}}{\tau_{C,v}}C_{v}^{i},\;\text{for}\;0<i<n_{C,v}$$

(7) $$\partial_{t}C_{v}^{n_{C,v}}=\frac{n_{C,v}}{\tau_{C,v}}C_{v}^{n_{C,v}-1}.$$

The variant-independent incidence is given by


(8) $$J_{C}=\sum_{v}\partial_{t}C_{v}^{n_{C,v}}=\sum_{v}\frac{n_{C,v}}{\tau_{C,v}}C_{v}^{n_{C,v}-1}.$$

For hospitalizations, we define


(9) $$\partial_{t}H_{v}^{0}=p_{H,v}f(t)[1-h_{v}(t)]S\alpha_{v}I_{v}-\frac{n_{H,v}}{\tau_{H,v}}H_{v}^{0}$$

(10) $$\partial_{t}H_{v}^{i}=\frac{n_{H,v}}{\tau_{H,v}}H_{v}^{i-1}-\frac{n_{H,v}}{\tau_{H,v}}H_{v}^{i},\;\text{for}\;0<i<n_{H,v}$$

(11) $$\partial_{t}H_{v}^{n_{H,v}}=\frac{n_{H,v}}{\tau_{H,v}}H_{v}^{n_{H,v}-1}.$$

Here, $h_{v}(t)$ is the population-averaged efficacy against hospitalization at time *t*. The number of new hospitalizations at time *t* is


(12) $$J_{H}=\sum_{v}\partial_{t}H_{v}^{n_{H,v}}=\sum_{v}\frac{n_{H,v}}{\tau_{H,v}}H_{v}^{n_{H,v}-1}.$$

To model the number of ICU beds occupied, we define distributions for both the transition between infection and ICU admission and the length of stay in ICU. The equations follow with a total ICU occupancy


(13) $$\partial_{t}W_{v}^{0}=p_{U,v}f(t)[1-u_{v}(t)]S\alpha_{v}I_{v}-\frac{n_{W,v}}{\tau_{W,v}}W_{v}^{0}$$

(14) $$\partial_{t}W_{v}^{i}=\frac{n_{W,v}}{\tau_{W,v}}W_{v}^{i-1}-\frac{n_{W,v}}{\tau_{W,v}}W_{v}^{i},\;\text{for}\;0<i<n_{W,v}$$

(15) $$\partial_{t}U_{v}^{0}=\frac{n_{W,v}}{\tau_{W,v}}W_{v}^{n_{W,v}-1}-\frac{n_{U,v}}{\tau_{U,v}}U_{v}^{0}$$

(16) $$\partial_{t}U_{v}^{i}=\frac{n_{U,v}}{\tau_{U,v}}U_{v}^{i-1}-\frac{n_{U,v}}{\tau_{U,v}}U_{v}^{i},\;\text{for}\;0<i<n_{U,v}$$

(17) $$\partial_{t}U_{v}^{n_{U,v}}=\frac{n_{U,v}}{\tau_{U,v}}U_{v}^{n_{U,v}-1},$$

(18) $$U=\sum_{v}\sum_{i=0}^{n_{U,v}-1}U_{v}^{i}.$$

### Population-wide vaccine efficacies

To find time-dependent population-wide VEs against infection $s_{v}(t)$, hospitalization $h_{v}(t)$, and requiring intensive care $u_{v}(t)$, we assume four basic vaccination states: VE 14 days after completion of first vaccination series *V*_2_, VE after waning *X*_2_, VE 7 days after booster vaccination (‘booster’, *V_B_*), and booster VE after waning *X_B_*. We define the mean times of VE attenuation as $\theta_{2}+\theta_{2}^{\prime }$ and $\theta_{B}+\theta_{B}^{\prime }$, that is we additionally use intermediate compartments $V_{2}^{\prime }$ and $V_{B}^{\prime }$ to ensure a realistic approximation of the actual waning time distribution, introducing a temporal buffer between vaccination and waning during which the initial protection is constant. Initially, the entire population is unvaccinated, *A *=* *1. We model the temporal transition from unvaccinated to vaccinated of different vaccination statuses for each variant as


(19) $$\partial_{t}A=-\nu_{2}(t-14d)$$

(20) $$\partial_{t}V_{2}=\nu_{2}(t-14d)-\frac{1}{\theta_{2}}V_{2}-\frac{\nu_{B}(t-7d)}{X_{2}+V_{2}+V\prime_{2}}V_{2}$$

(21) $$\partial_{t}V_{2}^{\prime }=\frac{1}{\theta_{2}}V_{2}-\frac{1}{\theta_{2}^{\prime }}V\prime_{2}-\frac{\nu_{B}(t-7d)}{X_{2}+V_{2}+V\prime_{2}}V_{2}^{\prime }$$

(22) $$\partial_{t}X_{2}=\frac{1}{\theta_{2}^{\prime }}V\prime_{2}-\frac{\nu_{B}(t-7d)}{X_{2}+V_{2}+V_{2}^{\prime }}X_{2}$$

(23) $$\partial_{t}V_{B}=\nu_{B}(t-7d)-\frac{1}{\theta_{B}}V_{B}(t)$$

(24) $$\partial_{t}V\prime_{B}=\frac{1}{\theta_{B}}V_{B}(t)-\frac{1}{\theta_{B}^{\prime }}V\prime_{B}(t)$$

(25) $$\partial_{t}X_{B}=\frac{1}{\theta \prime_{B}}V\prime_{B}(t).$$

Here, we use the rates of completed vaccinations $\nu_{2}(t)$ and booster vaccinations $\nu_{B}(t)$, which can be determined as the progressive differences of the respective cumulative number of vaccinations [16]. Note that we assume the onset of maximum protection at 14 days after completion of the first vaccination series and at 7 days after booster vaccination. *V* and V′ represent states of maximum immunity that were constant for a mean time θ+θ′ and decreased thereafter (state *X*) at rate 1/θ′. Given the equations above, we have $A+V_{2}+V_{2}^{\prime }+X_{2}+V_{B}+V_{B}^{\prime }+X_{B}=1$ at all times.

Let $e_{v,b,c}$ be a placeholder for different VEs against infection, symptomatic disease, severe disease requiring hospitalization, or severe diseases requiring intensive care with variant *v*, vaccination status *b* ‘2 doses’ and ‘boostered’, as well as waning status c (*c *=* *0 initially and *c *=* w* after attenuation). The population-averaged VE with respect to variant *v* is then given by and the VE for vaccinated-only individuals is


(26) $$\begin{array}{cc} e_{v}(t) & =e_{v,2,0}\left[V_{2}(t)+V\prime_{2}(t)\right]+e_{v,2,w}X_{2}(t)+\\ & +e_{v,B,0}\left[V_{B}(t)+V\prime_{B}(t)\right]+e_{v,B,w}X_{B}(t), \end{array}$$

(27) $${\tilde{e}}_{v}(t)=e_{v}(t)/[1-A(t)].$$

Note that the observable ‘*e_v_*’ serves as a placeholder for the temporal functions describing VE against infection *s_v_*, severe disease requiring hospitalization *h_v_*, and severe disease requiring intensive care *u_v_*.

Using this model of waning immunity, we want to devise a method to obtain the average initial VE, the waning time parameters *θ* and θ′ as well as the average waned VE from empirical temporally resolved VE curves. To this end, we make use of the simplified model and assume that every individual of a population becomes vaccinated at time *t *=* *0, that is ν(t)=δ(t) with initial conditions A(0)=1, V(0)=V′(0)=X(0)=0, or ν(t)=0, with initial conditions V(0)=1, A(0)=V′(0)=X(0)=0. Then, the average share of individuals in the ‘waned’ compartment is given by and therefore, the population-averaged sigmoidal decrease of VE from maximum immunity to its attenuated value follows the equation


(28) $$\partial_{t}A=-\nu (t)$$

(29) $$\partial_{t}V=\nu (t)-\frac{1}{\theta }V$$

(30) $$\partial_{t}V\prime =\frac{1}{\theta }V-\frac{1}{\theta \prime }V\prime$$

(31) $$\partial_{t}X=\frac{1}{\theta \prime }V\prime ,$$

(32) $$X(t)=\{\begin{array}{cc} 1-\frac{\theta }{\theta -\theta \prime }e^{-t/\theta }+\frac{\theta \prime }{\theta -\theta \prime }e^{-t/\theta \prime }, & \theta \neq \theta \prime \\ 1-\left(1+\frac{t}{\theta }\right)e^{-t/\theta }, & \theta =\theta \prime \end{array},$$

(33) $$Z(t)=e_{0}[1-X(t)]+e_{w}X(t).$$

Note that the mathematical description is agnostic with regards to the interpretation of *Z*(*t*) representing either a population-averaged decay of individual step functions whose times of change have been drawn from a distribution or every individual following the same average attenuation and such interpretations will therefore not be drawn here.

For every combination of efficacy type e∈{s,h,u}, variant v∈{d,o}, and vaccine status b∈{2,B}, we can now fit Equation (33) against empirical VE attenuation data to obtain the respective values of $e_{v,b,0},\;e_{v,b,w},\;\theta_{v,b}$, and $\theta_{v,b}^{\prime }$, which can then be used to compute population-averaged VEs from Equation (26), and Equations (19)–(25).

To do so, we used data from the meta-review by [17] only considering data explicitly reporting VE against Delta. Time intervals since the administration of the second vaccine dose and the computed VE within these intervals were extracted. The mean of each interval minus 14 days was used to define the day of VE since peak immunity after two doses. If only the beginning of a time interval was defined (e.g. starting 140 days after administration), then the length of the time interval was measured from the previous one (e.g. time interval 1: ‘days 105 to 139’, time interval 2: ‘days 140+’; time interval 2 was then assigned an upper bound of (140d+139d−105d)=174d). One study identified in the review, [18], refers to an additional time point that was also considered here. Also mentioned in [19] is a study by [20], from which data of VE against infection with Delta after two doses were extracted. Here, too, incomplete time intervals were fitted in a manner analogous to Feikin *et al.* [21] providing data on VE against symptomatic infection with both Delta and Omicron after two doses as well as after booster vaccination. Because this study specifies time intervals in weeks, it was assumed that the first interval (Weeks 2–9) corresponded to Days 14–63 and subsequent intervals (Weeks 10–14, 15–19) each corresponded to the next day of the previous interval until the end of the last week in the interval (e.g. Weeks 10–14 thus corresponded to Days 64–98). The same procedure was used for booster vaccination data and was implemented here as well. Unless otherwise defined in the study, the mean of each time interval minus 7 days (rather than 14 days) was used to define the day of VE since maximum immunity. Data on VEs against hospitalization and intensive care with Delta were taken from a study by de Gier *et al*. [22]. The UK Health Security Agency published data on VE against symptomatic infection with Delta and Omicron and against hospitalization with Omicron after two vaccine doses and after one booster [7]. Data on VE against infection with Delta and Omicron were extracted from Hansen *et al*. [13]. Because protection after booster vaccination was determined here only as a comparison with fully vaccinated persons, only data on VE after two doses were extracted. In addition, the 95% confidence intervals (CIs) of VEs were taken from all studies and used as weights in fits. Our study exclusively refers to data published by the end of 2021.

As described above, Equation (33) was fitted to these data per variant, vaccination status, and the target variable. Assumptions were made for VEs for which no data are available, with results shown in Fig. 2.

For the future course of the vaccination campaign, we assumed that no more initial vaccinations were administered. Furthermore, we assumed that the rate of daily booster vaccinations maintained its level achieved in late December, such that the cumulative number of booster vaccinations, following a sigmoid function, reached (i) the number of all initially vaccinated persons in 2021 and (ii) 80% of those (Fig. 3).

With these fits and assumptions, Equations (19)–(25) were integrated to obtain the population-averaged VEs in Equation (26), shown in Figs 4 and 5.

### Calibration of transmissibility of VOCs to growth rates in December 2021

To determine the transmissibility of the VOCs Delta and Omicron in Germany, we measured the respective growth rates of the variants in December 2021 using reported data and data from the *Deutscher Elektronischer Sequenzdaten-Hub* (DESH, German Electronic Sequence Data Hub) and applied analytical approximations to derive the transmissibilities.

The growth rate Λ_*v*_ of a variant *v* at time *t* is given by the largest eigenvalue of the Jacobi matrix of the ODE system from Equations (1–4) as where $\eta_{v}(t)=f(t)\alpha_{v}[1-s_{v}(t)]$ is the time-dependent infection rate of a variant modulated with time-dependent contact behavior *f*(*t*). Here, *S*(*t*) is the time-varying relative proportion of susceptibles and $s_{v}(t)$ is the population-wide VE against infection. From Equation (34), the base transmissibility of a variant is given by


(34) $$\Lambda_{v}(t)=-\frac{\beta_{v}}{2}-\frac{\omega_{v}}{2}+\frac{\sqrt{4S(t)\eta_{v}(t)\omega_{v}+\beta_{v}^{2}-2\beta_{v}\omega_{v}+\omega_{v}^{2}}}{2},$$

(35) $$\alpha_{v}=\frac{\left(\Lambda_{v}+\omega_{v}\right)\left(\Lambda_{v}+\beta_{v}\right)}{Sf\omega_{v}(1-s_{v})}.$$

**Figure 6:**
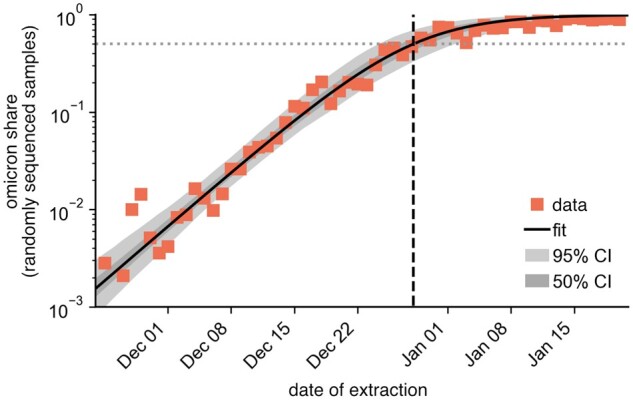
Omicron fixation dynamics with a numerical fit [sigmoid function, Equation (36)]. Data points represent the Omicron proportion in the random laboratory sample by date of extraction [28]. The dashed vertical line marks the time at which the Omicron proportion was 50% according to the fit. Additionally, we mark the 50% and 95% CIs of the fit.

From the fixation dynamics of Omicron (Fig. 6), a fit of the function to the measured proportion of Omicron in all new infections can be used to determine the fixation rate *μ*, which is related to the growth rates of the variants as


(36) $$\sigma (t)=\frac{1}{1+\text{exp}[-\mu (t-\tilde{t})]}$$

(37) $$\mu =\Lambda_{o}-\Lambda_{d}.$$

Hence follows on a calibration date *t*_1_.


(38) $$\begin{array}{c} \alpha_{o}={\left(4Sf(t_{1})[1-s_{o}(t_{1})]\omega_{o}\right)}^{-1}(-\beta_{o}^{2}+\omega_{o}\left(2\beta_{o}-\omega_{o}\right)\\ \;+(\beta_{o}-\beta_{d}+2\mu +\omega_{o}-\omega_{d}+\\ \;\;\;\sqrt{4S(t_{1})\eta_{d}(t_{1})\omega_{d}+\beta_{d}^{2}-2\beta_{d}\omega_{d}+\omega_{d}^{2}}{)}^{2}) \end{array}$$

The proportion of new infections attributed to the Omicron variant was measured from random laboratory samples of DESH data [23]. We assumed that the sample collection date corresponded to the date of symptom onset. We filtered collected samples by the ‘scorpio_call’ column, in which a sequenced genome is assigned to a VOC using the ‘Scorpio’ software (see descriptions in [24–26]). Here, all sequences whose ‘scorpio_call’ value contained the string ‘Delta’ were assigned to the VOC Delta, and all sequences whose ‘scorpio_call’ value contained the string ‘Omicron’ were assigned to the VOC Omicron (this included the value ‘probable Omicron’). In addition, filtering was done for sequences that were randomly selected for sequencing resulting in ${ℵ}_{d}(t)$ sequences of VOC Delta and ${ℵ}_{o}(t)$ sequences of VOC Omicron for each day. The proportion $\hat{\sigma }(t)={ℵ}_{o}(t)/[{ℵ}_{d}(t)+{ℵ}_{o}(t)]$ could then be fitted to the function Equation (36) with free parameters *μ* and $\tilde{t}$ (time at which Omicron would account for 50% of new infections). The fit was performed with Markov chain Monte Carlo sampling to minimize the sum of residuals in logarithmic space $\sum_{t\prime }\left[\text{log}\;\sigma (t\prime )-\text{log}\;\hat{\sigma }(t\prime )\right]$, with 100 walkers and 1000 steps each. We thus obtained an ensemble of 100 000 parameter pairs $\tilde{t}$ and *μ*. We found mean values of $\langle \mu \rangle =(0.184\pm 0.019)d^{-1}$ and $\langle \tilde{t}\rangle -t^{\left(o\right)}=(35.2\pm 2.4)d$, where $t^{\left(o\right)}=\text{November}\;23,\;2021$ (date of collection of the first Omicron samples).

To determine the growth rate of Delta, we used the number of new infections after symptom onset, imputed using a nowcasting technique [27], and restricted ourselves to the period between 1 December 2021 and 15 December 2021. Let ${\hat{J}}_{S,\text{tot}}(t)$ be the number of new infections after symptom onset on date *t*. Then ${\hat{J}}_{S,d}(t)={\hat{J}}_{S,\text{tot}}(t)[1-\sigma (t)]$ is the number of daily new infections with Delta (with mean values of *μ* and $\tilde{t}$). In this way, we transformed the measured total new infections and fit an exponential decrease $J_{s}(t)$ to ${\hat{J}}_{S,d}(t)$.

The doubling time of a variant was measured as resulting in 4.5–5.5 days for Omicron cases in December 2021.


(39) $$T_{\text{dbl},v}(t)=\frac{\ln2}{\Lambda_{v}(t)},$$

By Equation (35), the ratio of the base transmissibility of two variants is given as


(40) $$\frac{\alpha_{o}}{\alpha_{d}}=\frac{1-s_{d}}{1-s_{o}}\times \frac{\left(\Lambda_{d}+\mu +\omega_{o}\right)\left(\Lambda_{d}+\mu +\beta_{o}\right)}{\left(\Lambda_{d}+\omega_{d}\right)\left(\Lambda_{d}+\beta_{d}\right)}.$$

In the special case of constant latency and infectious period, as well as $s_{d}\equiv s$ and $s_{o}\equiv (1-\epsilon )s$ with ‘immune evasion’ *ϵ*, we find


(41) $$\frac{\alpha_{o}}{\alpha_{d}}=\frac{1-s}{1-(1-\epsilon )s}\left(1+\frac{\mu }{\Lambda_{d}+\omega }\right)\left(1+\frac{\mu }{\Lambda_{d}+\beta }\right).$$

From this equation, it can be seen that both smaller latency periods (larger *ω*) and smaller infectious periods (larger *β*) require smaller increases in base transmissibility to explain the observed rates Λ_*d*_ and *μ*. For example, assuming *s *=* *0.5, $\epsilon =0.9,\;\mu =0.19/d,\;\Lambda_{d}=-0.045/d$, and ω=1/2d, we obtain a transmissibility increase of ${\left(\alpha_{o}/\alpha_{d}\right)}_{1}=100\%+119\%$ for $\beta_{1}=1/7d$ and an increase of ${\left(\alpha_{o}/\alpha_{d}\right)}_{2}=100\%+24\%$ for $\beta_{2}=1/3d$, which illustrates the implausibility of longer infectious periods.

### Calibration of the contact modulation

The transmissibility of Delta *α_d_* was chosen such that $R_{0}=\alpha_{d}/\beta_{d}$ with fixed *R*_0_. The exact value of *R*_0_ played a minor role due to the freely chosen contact modulation *f*(*t*) (only the scale of *f*(*t*) was determined by *R*_0_—a high *R*_0_ required a lower *f*(*t*) to explain the observed rates than a lower *R*_0_). The contact modulation at time *t* was derived from Equation (34) to be


(42) $$\hat{f}(t)=\frac{\left({\hat{\Lambda }}_{d}(t)+\beta_{d}\right)\left({\hat{\Lambda }}_{d}(t)+\omega_{d}\right)}{\hat{S}(t)R_{0}\beta_{d}\omega_{d}[1-s_{d}(t)]}.$$

We determined the empirical growth rate by the growth of the incidence of Delta cases ${\hat{J}}_{C,d}=[1-\sigma (t)]{\hat{J}}_{C}$ (here, ${\hat{J}}_{C}$ is the 7-day average of total incidence by reporting date and σ(t) is the fitted fixation curve of the VOC Omicron), obtained from the identity $\Lambda_{d}(t)=\partial_{t}\text{ln}{\hat{J}}_{C,d}$ by in the discrete approximation with Δt=1d and with reporting delay $t_{\text{shift}}$. A value of $t_{\text{shift}}=7d$ satisfactorily approximated the reporting delay in practice. This value was chosen because, for a delta-peak incidence at time *t *=* *0, the reported incidence will be equal to its associated waiting time distribution, with the corresponding peak occurring at the distribution’s mode, which for the Erlang distribution is given by $\approx (n_{C}-1)\tau_{C}/n_{C}\approx 7d$.


(43) $${\hat{\Lambda }}_{d}(t)=\frac{1}{\Delta t}\text{ln}\left(\frac{{\hat{J}}_{C,d}(t+1+t_{\text{shift}})}{{\hat{J}}_{C,d}(t+t_{\text{shift}})}\right)$$

For the share of susceptibles at time *t*, we chose with the proportion of recorded cases *p_C_*.


(44) $$\hat{S}(t)=1-\frac{1}{Np_{C}}\sum_{t\prime =0}^{t}{\hat{J}}_{C}(t\prime +t_{\text{shift}})$$

The remaining free parameter for model calibration was the proportion of initially infected *I*_0_. We used the values shown in Table 2.

**Table 2:** Initial conditions with $t_{0}=\text{Jul}\;1,\;2021$ for different values of latency and infectious period.

**Latency** $\omega^{-1}$ **[d]**	**Inf.per.** $\beta^{-1}$ **[d]**	$I_{0}/10^{-5}$
1	2	5.8
2	2	7.2
1	3	7.7
2	3	9.0

### Extrapolation of the contact modulation

Similar to the procedure in [28], we assumed that the empirically found contact modulation $\hat{f}(t)$ according to Equation (42) followed a stochastic process with autocorrelation time $\vartheta^{-1}$ and extrapolated the series based on an Ornstein–Uhlenbeck process as


(45) $$df_{t}=\vartheta (f_{m}-f_{t})dt+\xi dW_{t}.$$

The process generates a time series *f_t_* with mean *f_m_* and variance $\xi^{2}/(2\vartheta )$, using a Wiener process $dW_{t}$. The autocorrelation time was obtained from the empirical curve $\hat{f}$ as approximately $\vartheta^{-1}=21d$. We chose $t_{\text{end}}=184d$ (1 January 2022) as the start of the extrapolation time. To determine *f_m_* and *ξ*, we measured the mean $f_{m}=\langle \hat{f}\rangle$ and the variance Var[f] in the period t∈[150d,184d]. The initial condition at time $t_{\text{end}}$ was set to $f_{t}=\hat{f}(t)$, then the equation was integrated with Δt=0.1d and sampled with Δt=1d. The choice of the calibration end date had an impact on the course of the wave in early January 2022. Since the model course with $t_{\text{end}}=184d$ satisfactorily reflected the empirical data in January 2022, this value was not changed retrospectively.

The continuous function *f*(*t*) required for model integration was obtained from linear interpolation of the tabulated values of $\hat{f}(t)$ and *f_t_* (tabulated for individual days).

**Figure 7:**
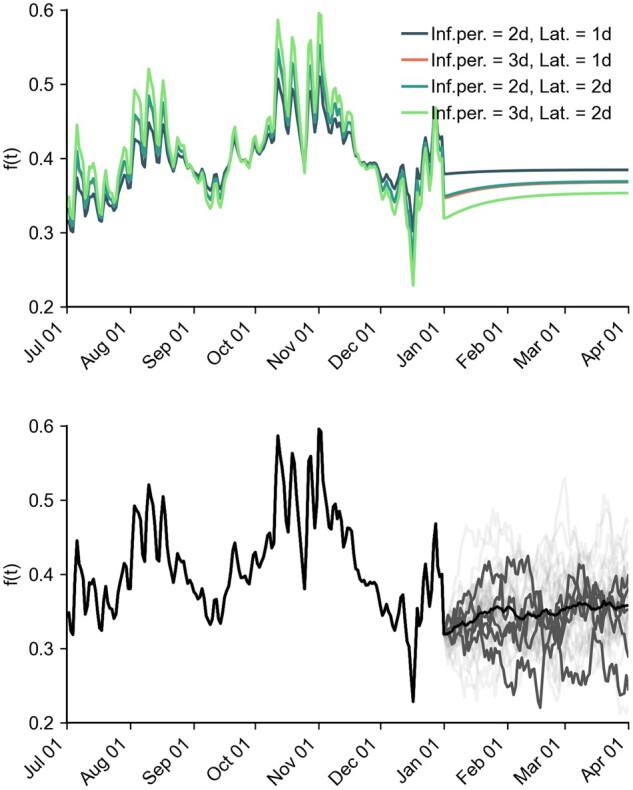
Time courses of contact modulation. (**a**) Contact modulation for different generation times, inferred until 1 January 2022, then deterministically continued according to Equation (45) with *ξ *= 0 (all curves for ‘medium reach’ and ‘low VE’). (**b**) Example stochastic extrapolations of contact modulation according to Equation (45) for ‘medium reach’, ‘low VE’ and a generation time of 5 days (2 days latent + 3 days infectious period). The black curve shows the mean, dark gray curves show five randomly selected trajectories and light gray curves show additional trajectories.

Example runs for individual simulations are shown in Fig. 7.

### Latency and infectious period

The time scales relevant for SEIR models are latency $T_{L}=1/\omega$ and infectious period $T_{I}=1/\beta$, which sum to generation time $T_{G}=T_{L}+T_{I}$ [29, 30].

We chose the latency period of VOC Delta as $T_{L}=2d$, resulting from an incubation period of ∼4 days [31] and the observation that the infectious period for the wild-type began, on average, 2 days before symptom onset [32]. One study from the UK [33] assumed a latency period of 2.5 days for both the wild-type and the VOC Alpha.

Another study from the UK found a mean generation time of ∼5 days for VOC Delta [34], composed of a latency period of ∼1 day, a presymptomatic infectious period of ∼3 days, and a symptomatic infectious period of 1 day. To correspond to the generation time found in this way without changing the assumptions regarding the latency period, we assumed an infectious period of 3 days (2 days pre-symptomatic and 1 day symptomatic) as a lower limit. In previous analyses for Germany, a shorter generation time of 4 days was assumed for VOC Delta, among others, which we have therefore also included in our analyses as a plausible value [27]. Note that we considered longer infectious periods implausible as per our estimations based on Equation (41).

Due to limited data, we assumed in a first analysis that the VOC Omicron was associated with the same values for *T_L_* and *T_I_* as the VOC Delta. However, initial observations suggested that Omicron has a shorter serial interval (2.2 days on average in the Republic of Korea [35]) than Delta (3 days on average in the Republic of Singapore [36]). In the SEIR model, the mean serial interval is equal to the mean generation time [29]. We therefore assumed for additional analyses that Omicron has a shorter latency of only $T_{L}=1d$.

Note that in later published studies, a mean serial interval of 4 days for Delta [37], respectively, of 4.2 days for Delta and 3.6 days for Omicron was found for household clusters in Germany [38], which is in line with our estimations of the generation time.

At the time, no other reliable estimates for the mean latency and infectious period in Germany existed. We therefore decided to iterate plausible combinations of these parameter values instead of drawing their values from distributions that would be pure assumptions, as well. Doing so comes with the additional advantage that as soon as more reliable estimates exist, adjusted modeling results can be easily estimated by interpolation from previous results.

### Remaining parameters

We assumed a reporting rate of 50%, that is every second infection was reported. This parameter was informed by seroprevalence analyses [39], where a detection rate of 55% was found for individuals older than 17 years. As the ratio of asymptomatic infection was reported to be higher for the younger population for previous variants [40], we assumed a slightly lower population-wide ascertainment of 50%. In retrospect, this assumption remains plausible [41]. While different opinions prevail regarding the influence of high incidences on ascertainment, we found that constant ascertainment reflected the temporal interplay of reported incidence, hospitalization rate, and ICU occupancy reasonably well. To fit our model to the 7-day average incidence, we chose an Erlang distribution with *n_C_* = 3 and $\tau_{C}=11d$ between infection and reporting. Here, we mapped the incubation period of ∼4 days, plus a reporting delay of 4 days [27] in addition to a 3-day systematic shift by the moving average. We took the 7-day average of new infections per day from ref. [42].

The number and immunity of recovered individuals were, at the time of analysis, unclear. A substantial number of recovered individuals was expected to have been vaccinated, and ergo part of the vaccinated population. We assumed that the immunity of the recovered decreases over time. Therefore, we calibrated the model to follow the Delta wave in the fall of 2021 and assumed that those recently recovered had full immunity against infection with Omicron, but that the unvaccinated recovered population from the first three pandemic waves had no protection against infection with Omicron unless they had been vaccinated additionally. For this reason, we implemented the model starting 1 July 2021, with no recovered individuals initially. Retrospectively, we find that from the ≈3.7 mio cases reported until 1 July 2021, 74% likely received a vaccination according to survey studies [43–45] and are therefore considered in our analysis as part of the vaccinated population (through the population-averaged VE). With an ascertainment ratio of about 50%, we estimate that the total number of nonvaccinated recovered individuals was on the order of 2.4% of the population in Germany at the time. Following the same reasoning, from the ≈3 mio cases that were reported as infected with the Delta variant in the following wave, a percentage of similar order was counted as entirely immune to infection with Omicron in our model. As both percentages are of comparable size, it is reasonable to assume that both assumptions balance each other out and are of small effect nonetheless.

**Figure 8:**
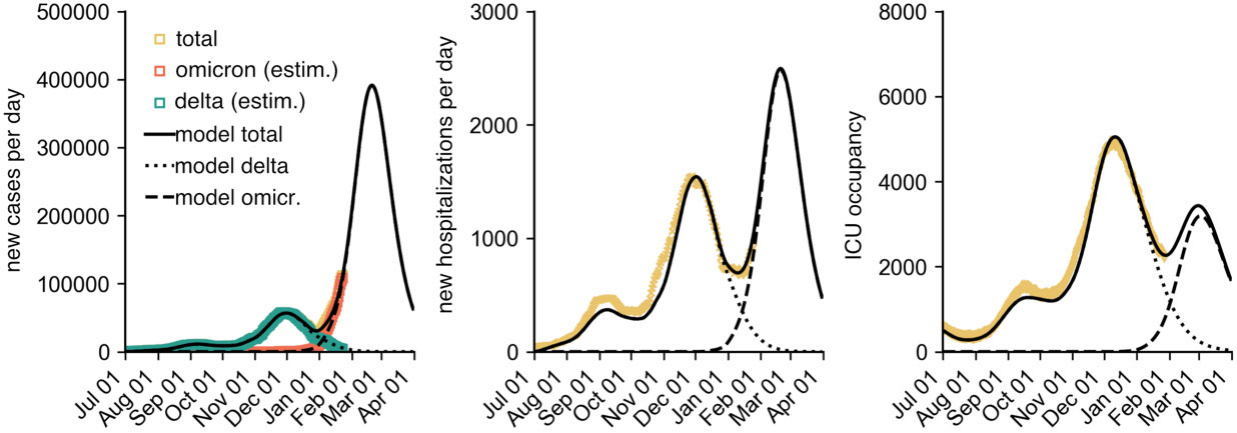
Calibration of the model to the fall of 2021 Delta wave assuming a mean latency and infectious period of 2 days each for Delta and 1 day and 2 days, respectively, for Omicron. This calibration further assumed that all of those fully vaccinated by the end of 2021 received a booster vaccination (high reach) and that VEs match the data in Fig. 2. In addition, the RR of hospitalization due to Omicron versus Delta was assumed to be *RR *=* *0.35 and intensive care *RR *=* *0.15.

For the number of daily new hospitalizations, we assumed a hospitalization probability of $p_{H,d}=2.0\%$ for unvaccinated persons, and a probability of intensive care of $p_{U,d}=0.45\%$ (both values were per reported case, not per infection). We set the length of stay in an ICU equal to the observed length of stay observed during the first pandemic wave at $\tau_{U}=18d$ and set *n_U_* = 3 to represent the observed median and interquartile range (IQR) sufficiently accurately (C. Karagiannidis, personal communications). Length of stay correlated strongly with ICU probability, so different pairs of values of these two parameters could generate rather similar trajectories of ICU occupancy. We chose the transition time from infection to hospitalization as $\tau_{H}=13d$ with *n_H_* = 2 and the transition time between infection and intensive care as $\tau_{W}=14d$ with *n_U_* = 1. We took the 7-day average of new hospitalizations per day from ref. [46] (adjusted time series) and the ICU occupancy in Germany from ref. [47]. The above values were chosen in a way that the model curves depicted the course of the Delta wave well (see as an example Fig. 8).

We set N=83155031 as the total population [46].

### Contact reduction

To investigate the influence of different contact reductions on the course of the wave, we simulated seven different scenarios. In the base scenario, contact modulations *f*(*t*) were not changed. For reductions, the curve was scaled between two time points $t_{r,0}$ and $t_{r,1}$. This resulted in the modified contact modulation with 0≤r≤1. We chose values of (i) r=20%, from 31 January to 15 March, (ii) r=50%, from 31 January to 15 February, (iii) r=50%, from 31 January to 28 February, (iv) r=50%, from 31 January to 15 March, (v) r=50%, from 15 February 15 to 15 March, and (vi) r=50% from 1 January to 15 January. Example model runs for contact reductions are shown in Fig. 10 and Supplementary Fig. 3.


(46) $$f_{r}(t)=\{\begin{array}{cc} \left(1-r)f(t\right) & t_{r,0}\leq t\leq t_{r,1}\\ f(t) & \text{else} \end{array}$$

### Model simulations

The model was implemented and analyzed using the simulation software *epipack* [48]. As initial conditions for $t_{0}=\text{Jul}\;1,\;2021$, we chose the values shown in Table 2. The model was integrated up to $t_{1}=\text{Dec}\;1,\;2021$, using a Runge–Kutta 4(5) method with dynamic step size control. We assumed an initial Omicron share of $\sigma (t_{1})$ and fixed the modified initial conditions to $I_{d}^{\text{new}}(t_{1})=[1-\sigma (t_{1})]I_{d}(t_{1})$ and $I_{o}^{\text{new}}(t_{1})=\sigma (t_{1})I_{o}(t_{1})$ (all other compartments were assigned the respective values they assumed in the final state of the previous integration). Finally, the model was integrated until $t_{2}=\text{Apr}\;1,\;2022$. An example integration including the calibration based on the Delta wave is shown in Fig. 8.

## Results

The purpose of our analyses was to provide, at the time, order-of-magnitude estimates of central epidemiological observables and scenario comparisons with regard to variations in parameters that determined the course of the Omicron infection wave.

Combining plausible assumptions and stochastic extrapolations of the contact modulation, we expected a maximum median of 300 000 new cases per day, associated with a wide uncertainty of 180 000 or 450 000 cases per day (50% PI) and 55 000 or 800 000 per day (95% PI) (see Fig. 1 and Table 1). Overall, a median outbreak size (cumulative number of reported Omicron cases) of 16.5 million was expected by 1 April 2022 (50% PI: 11.4–21.3, 95% PI: 4.1–27.9). This figure was expected to be an overestimation at the time, as the reported cases might have been artificially reduced by changes in test prioritization or exhaustion of reporting logistics capacities, and the outbreak size is systematically overestimated due to modeling choices. Note that the model was calibrated up to 1 January 2022. Retrospectively, approximately 14.8 million Omicron infections have been reported up to 1 April 2022 [23, 41], which is ∼10% below our median expectation; hence, rather accurate considering we expected our model to overestimate the peak size by a maximum of 10%. Similarly, the observed incidence peaks in mid-February (peak: 191k daily new cases) and mid-March (peak: 230k daily new cases) [23], as well as the time series of new hospitalizations and cases in ICU care, respectively, lied within expectation.

Regarding the temporal evolution of the outbreak, several of the simulated outbreaks had two peaks as was then later observed in the actual outbreak, the latter peak likely being caused by the spread of the BA.2 sublineage of Omicron, which is associated with a higher base transmissibility [49]. However, most of the simulated outbreak, as well as the time series of the scenario median only shows a single peak. This scenario median time series first overestimated the daily number of new infections (in February) and subsequently underestimated them (in March).

**Figure 9:**
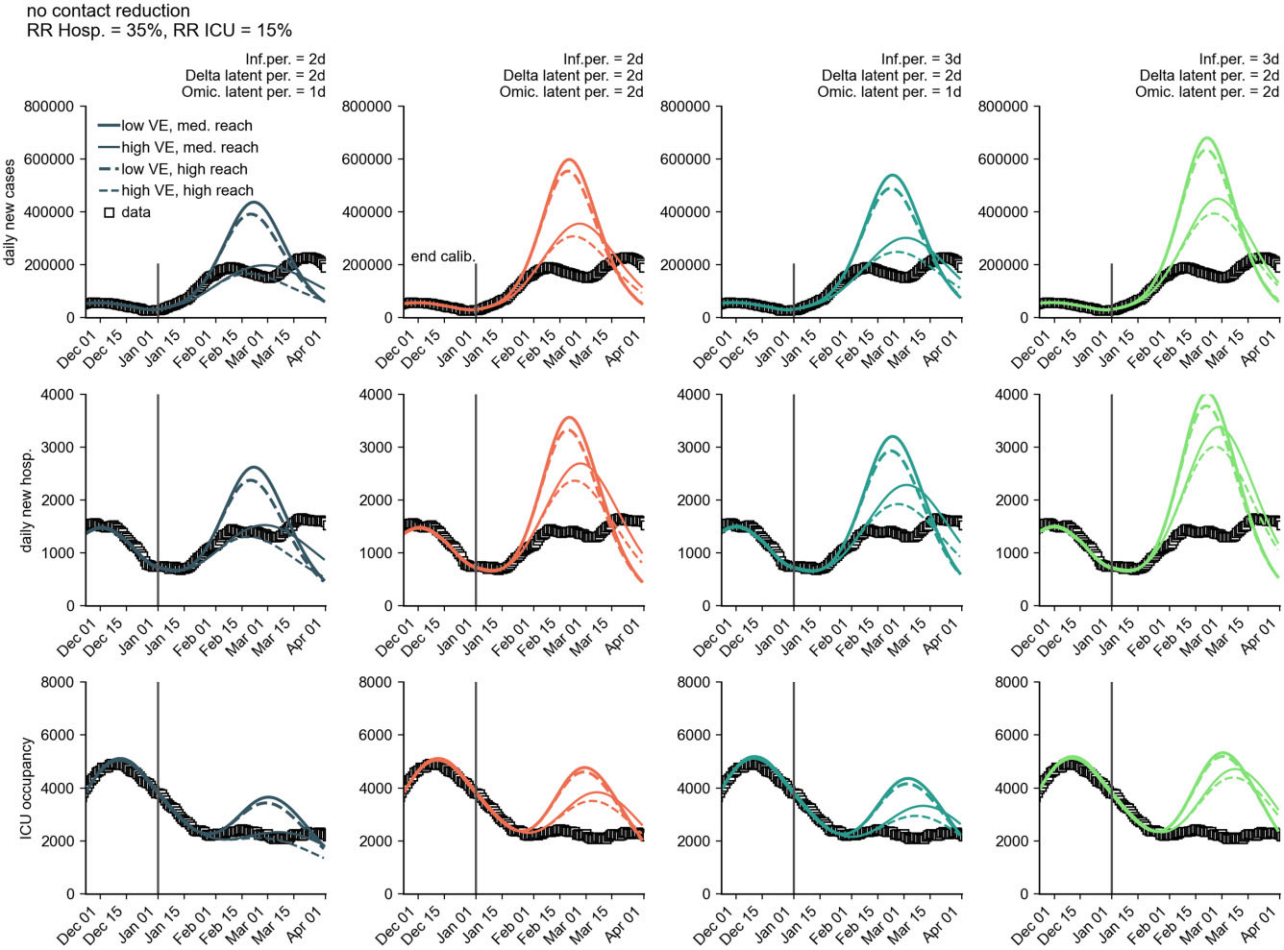
Influence of generation time on model results for the number of new cases per day (first row), new hospitalizations per day (second row), and ICU occupancy (third row) for a combination of different plausible model assumptions. Iterated here were ‘medium reach’ and ‘high reach’ of the booster campaign, ‘low VE’ and ‘high VE’ of the booster vaccination, as well as different generation times of Omicron [5 days, 4 days (Omicron latency: 2 days), 4 days (Omicron latency: 1 day), and 3 days]. We defined the latency period as the mean duration between infection and the onset of infectiousness. It was further assumed that no additional contact reduction occurs. For each scenario combination, a simulation was performed with an average course of the contact behavior (stochastic simulation with zero variance). Furthermore, the RR of hospitalization by Omicron versus Delta was assumed to be *RR *=* *0.35 and intensive care *RR *=* *0.15.

The results were sensitive to variations in the assumed generation time (Fig. 9). Small generation times caused larger growth rates with constant transmissibility of a variant (see ‘Calibration of transmissibility of VOCs to growth rates in December 2021’ section). This meant that rapid increases in case numbers must be attributed to a higher basic reproduction number for longer generation times, which, in turn, caused larger model outbreaks than pathogens with shorter generation times, but the same growth rate. Model results of different generation times and assumed VE of the booster vaccine reflected, at the time, the observed data similarly well, such that the analysis presented did not allow for conclusions about the actual contribution of the booster vaccination to the incidence. Retrospectively, the ‘medium reach’ and ‘high VE’ scenarios show good agreement with observed data (see Supplementary Material Section 1.2 and Supplementary Fig. S2).

The model also showed a high sensitivity to the assumed booster VE against infection. The booster vaccine reach is less conclusive (within the range of 80–100% of initially immunized individuals).

**Figure 10:**
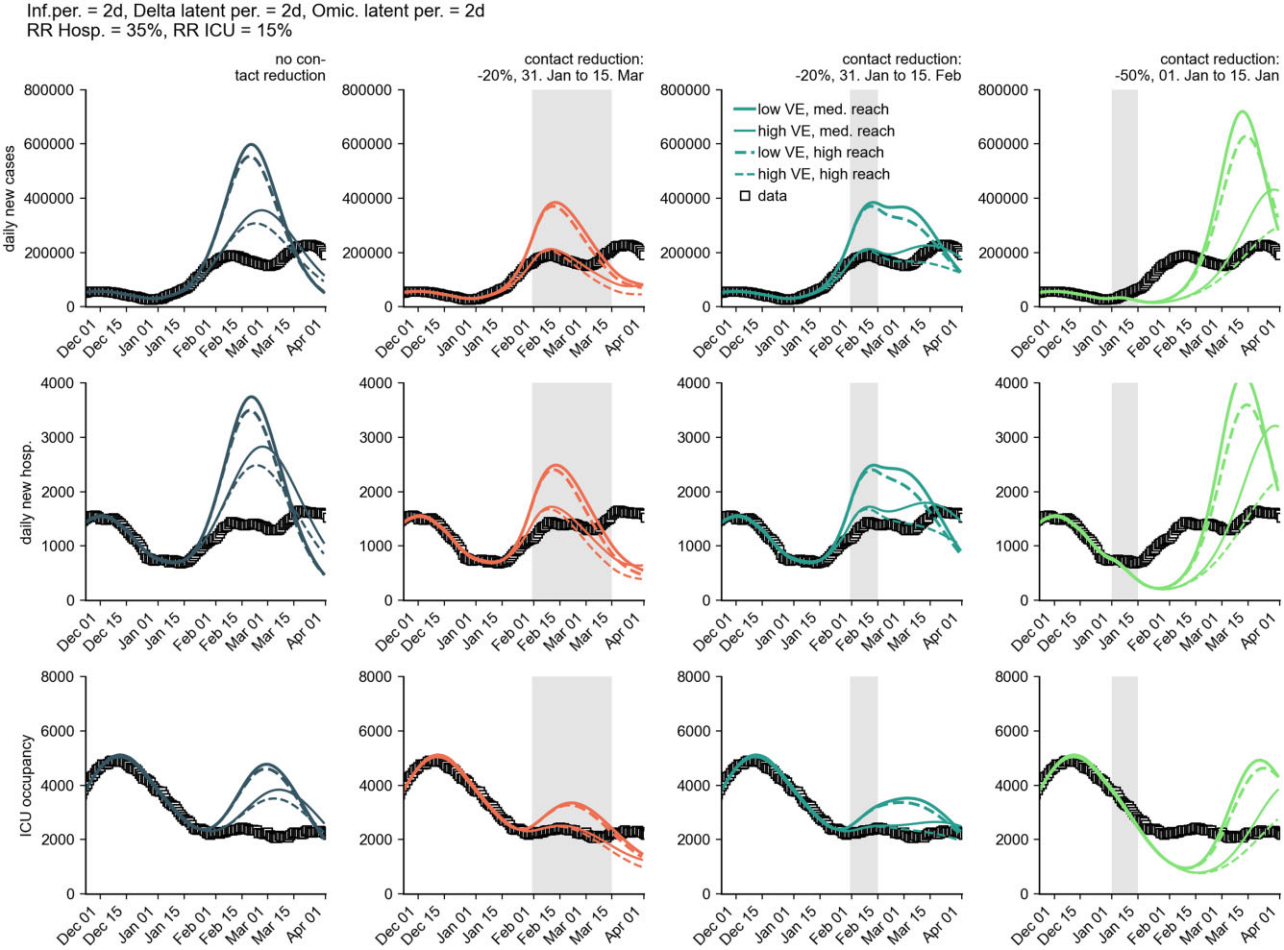
Comparison of model results for different contact reductions. An early, strong contact reduction could have led to a strong rebound effect (right column). A slighter, long contact reduction (–20% by 15 March) led to a smaller outbreak (by 1 April, second column from left). However, this could have been followed by another smaller wave after the end of the contact reduction period (data not shown here due to uncertainties in the forecast horizon beyond March). A slight, short contact reduction led to a flattening of the infection wave and thus also to containment (second column from right) with a sustained continuation of systemic immunity by infection. Shown are the results for the number of new cases per day, new hospitalizations per day, and ICU occupancy for a combination of different model assumptions. Iterated here were ‘medium reach’ and ‘high reach’ of the booster campaign, ‘low VE’ and ‘high VE’ of the booster vaccination, as well as various contact reductions. Here, a generation time of 4 days was assumed for both variants (2 day latency). For each scenario combination, a simulation was performed with an average course of the contact behavior (stochastic simulation with zero variance). Furthermore, the RR of hospitalization by Omicron versus Delta was assumed to be *RR *=* *0.35 and intensive care *RR *=* *0.15.

**Figure 11:**
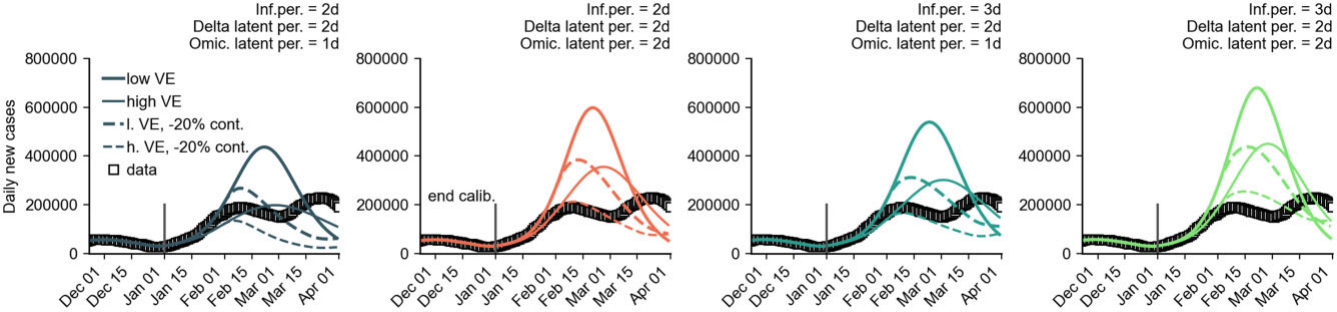
Contact reduction of –20% compared to the original trajectories for all generation times. Shown are the results for the number of new cases per day for a combination of different model assumptions. ‘Medium reach’ of the booster campaign, ‘low VE’, and ‘high VE’ of the booster vaccination were also iterated here. For each scenario combination, a simulation was performed with an average course of the contact behavior (stochastic simulation with zero variance). Furthermore, the RR of hospitalization by Omicron versus Delta was assumed to be *RR *=* *0.35 and intensive care *RR *=* *0.15.

Variations in contact behavior can have a significant impact on the results, as well. Slight reductions in contact, such as those brought about by autonomous changes in the behavior of the population [14], led to substantial reductions in outbreak size in the model (Figs 10 and 11). Potentially, an additional wave could have been expected after the end of the model integration phase. This wave should have, however, been smaller due to the basic immunity in the population achieved by the first wave. This effect is illustrated by a weak, short contact reduction (–20% from 31 January to 15 February), which would have had a ‘breaking’ effect on the wave and leads to a flattening of the epidemic curve over a longer period of time. However, early, strict, yet short contact restrictions could have led to a ‘rebound’ effect (Fig. 10) due to a lack of population-wide immunity to infection. Such an NPI would have caused larger outbreak sizes since the population-wide effect of the booster vaccination against infection would have already diminished by then (Fig. 4).

For unchanged contact behavior, we found a maximum permissible RR of requiring intensive care in the range of 10–20% in order to keep ICU occupancy below a critical value of 4800 beds (Supplementary Tables S1–S12).

Results for cumulative outbreak sizes and maxima of incidence, hospitalization incidence, and ICU occupancy are shown in Supplementary Tables S5–S12.

**Figure 12:**
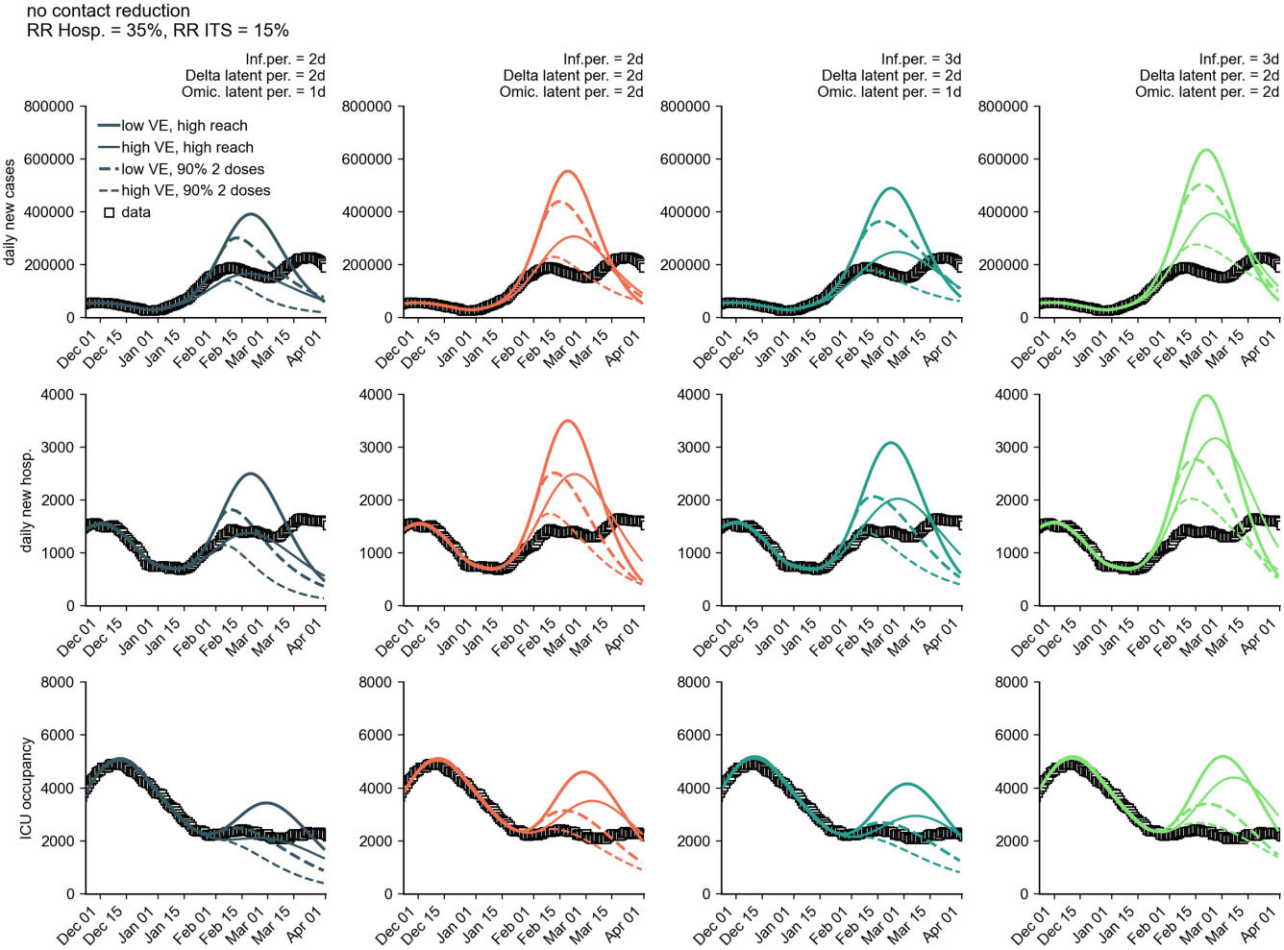
Comparison of model trajectories if, as of 22 January 2022, 15 million previously unvaccinated persons would have the same level of immune protection as after completion of the initial vaccination series. Shown are the number of new cases per day (first row), new hospitalizations per day (second row), and ICU occupancy (third row) for a combination of different model assumptions (see Materials and methods section). The solid curves correspond to the results of the ‘high reach’ scenario from Fig. 1 and the dashed curves to the corresponding scenario of an initial immunization rate of ∼90%.

The hypothetical case of a short-term, drastic increase in the initial immunization rate illustrated the contribution to the pandemic of those who were still unvaccinated (Fig. 12). Here, it was assumed that, as of 22 January 2022, 15 million previously unvaccinated individuals would have achieved the initial full immunization status. A high initial immunization rate would have resulted in a large reduction in ICU burden due to the high efficacy of the vaccines against severe courses.

Retrospectively, our results agreed well with the scenario ‘medium reach’, ‘high VE’, and –20% contact reductions (a short time in early February 2022) for a mean latent period of 4 days and a mean infectious period of 4 days for both variants, cf. third column from the left in Fig. 10. The ‘medium reach’ assumption of administering booster vaccinations to 80% of the people who had completed first vaccination series proved to be an accurate estimation. Furthermore, the temporal evolution of the VE in the ‘high VE’ scenario shows satisfying agreement with the time series of VE that was estimated using Farrington’s method counting breakthrough infections, see Supplementary Fig. S2 (with the data containing mostly symptomatic infections, however, which hinders a definite and direct comparison). The short decrease in growth rate forced by a 20% reduction in the contact modulation led to a peak size of ∼200 000 new cases per day in the model, followed by a resurgence and a slightly larger second peak, both of which reflects the actual time series of the outbreak quite well. Since no decrease in contact or mobility behavior was observed in Germany during this period of time, however, the first decrease in the growth rate has probably been induced by other factors and was likely caused by an incipient systemic immunity, which implies that we might have underestimated the initial systemic immunity against infection with the Omicron variant. The second peak was, with high certainty, caused entirely by the spread of the BA.2 sublineage of Omicron, which was associated with even higher base transmissibility and therefore led to a net increase in growth rate after the initial drop. The combination of these two effects has likely led to a temporal evolution of the growth rate that was reflected well by the ‘–20% contact reduction for a short time’ scenario in the model.

## Discussion and conclusions

The presented results are subject to a number of limitations due to both model structures, as well as various uncertainties in the assumptions. For example, population-averaged models that do not explicitly distinguish between vaccinated and unvaccinated individuals can systematically overestimate the size of major outbreaks by an order of magnitude of ∼10%. We therefore expected the actual outbreak size to be lower than those reported here. Nevertheless, we decided to not explicitly make a distinction between vaccinated and unvaccinated individuals, enabling us to adjust, simulate, and reanalyze the model in a dynamic manner. This facilitated an agile response to changes in data—an advantage that justified uncertainties and allowed for sustainable analysis procedures due to the structural stability of the model. By comparing many different scenarios, it was thus possible to quickly analyze and illustrate which aspects of the expected dynamics are robust to parameter changes and to which the model reacted sensitively to.

Further systematic overestimation of outbreak sizes may result from assumptions about the contact structure. In the present case, a homogeneous contact structure was assumed, which is the same within and between all age groups of the population. This simplification, which is not met in reality, likely leads to an overestimation of the number of cases since heterogeneities in the contact structure of age groups usually lead to lower outbreak sizes [50]. Furthermore, this effect may have led to an underestimation of the RR of hospitalization and requiring intensive care, as the dynamics at the beginning of a wave are often dominated by younger age groups, which are usually at lower risk of severe disease. As more elderly people become infected, for whom the probability of a severe course is higher than for younger people, the at-the-time observed RR may have increased again and, with it, ICU occupancy. However, as with the distinction in vaccination status, a heterogeneous contact structure was also disregarded in favor of reducing model complexity.

The average contact behavior in December 2021 was chosen as the basis for extending the contact modulation. At the time, it was reasonable to assume that this contact behavior could be extended into January 2022 and the following months due to then-unchanging protective measures. Since the contact behavior was not at pre-pandemic levels, it could be assumed that increased contact behavior would be observed by the end of the first Omicron wave at the latest, which could lead to another wave. Due to large uncertainties as to when an increased contact behavior could be expected, to what extent this increase would occur, uncertainties as to how long an Omicron infection protects against re-infection, uncertainties regarding a possible underreporting of infections, as well as the influence of seasonality on the spread of Omicron, this effect was not considered here. In this sense, an underestimation of the number of cases beyond 1 April 2022 was to be expected.

Furthermore, the concept of a possible ‘automatic emergency brake’ was disregarded in this study, that is no automatic contact reduction was to be implemented as soon as case numbers, hospitalizations, or ICU occupancy exceeded a critical value. However, we illustrated this effect by the influence of a long, weak contact reduction of –20%.

There are also other possible limitations due to uncertainties in the assumptions made or the processes underlying the model:

- At the time of model development in mid-December 2021, there was uncertainty about the immunity of recovered individuals to reinfection with Omicron. In the model, all persons who recovered from the first three pandemic waves were assumed to be susceptible (except vaccinated recovered individuals, who were equated with vaccinated persons that were not previously infected), while those who recovered from the Delta wave were assumed to be 100% immune. This results in an infection-induced initial systemic immunity that lies between these two extremes. In reality, increased immunity of those infected from earlier waves could lead to an overestimation of peak heights in the model. In the same sense, reduced immunity after a Delta infection or decreasing immunity over time, not taken into account here, could be the cause of an underestimation of the peak heights. Mathematically, our approach only served the purpose of assuming a certain, comparatively low, basic immunity against infection with Omicron in the population, that is the above-mentioned model assumptions cannot be easily transferred to reality. However, due to the unclear data regarding the immunity of recovered persons with respect to infections with Omicron during the development of the model in mid-December 2021, our approach can be considered feasible at the time.
- Similarly, there was uncertainty in the number of recovered persons who received a vaccination after infection and, thus, achieved at least primary immunization status. Since recovered individuals from the first waves were assumed to have no immunity (see above) and treated the same as susceptibles, we implicitly assumed a vaccination rate of recovered individuals equal to the population-wide vaccination rate. Given the unclear data situation during model development in mid-December 2021, this approach can also be considered practicable. Retrospectively, this assumption was justified by survey studies that asserted that 74% of the recovered individuals would vaccinate too [43–45].
- With regard to the exact estimation of the expected number of cases, it should be noted that the assumed under-ascertainment (i.e. the proportion of unreported infections) was also subject to uncertainty and could only be roughly estimated. In the present case, we assumed a constant reporting rate of 50%, that is every second infection was reported. If the number of unreported infections was higher in reality, then the observed case numbers were likely to be smaller than those estimated by the model because natural population-wide immunity would be achieved earlier at the then-current level of contact behavior (effective *R*-value of $R_{\text{eff}}<1$). Similarly, underreporting may have further increased in the following months due to changes in the prioritization of testing or other logistical limitations. Conversely, a relaxation of the situation could have led to a subsequent increase in ascertainment during the decline of an epidemic wave and therefore a decay in the reported number of infections that is slower than the true decline. At the time, however, a constant reporting rate of 50% seemed a plausible assumption [40].

The VE against infection with Delta assumed in this analysis was approximately equal to the VE against symptomatic disease with Delta, a result of regression of the collected data on VEs. However, because many studies estimated VE against infection to be lower than VE against symptomatic disease, we may have been overestimating the efficacy of vaccines against infection with Delta. Furthermore, ignoring the VE of the AstraZeneca and Johnson & Johnson vector vaccines also leads to a systematic, though likely not considerable, overestimation of the VE against infection with Delta. In the case of VE overestimation, Omicron growth would have been driven more by an increase in base transmissibility, as immune evasion would be lower. Since higher transmissibility leads to greater outbreaks, this would have implied an underestimation of outbreak size in our results. However, the model also ignored VE against transmission, which has been observed to be non-negligible in several countries. This VE against transmission would again raise the effective contribution of vaccination to the attenuation of the incidence [51]. In retrospect, we seem to not have overestimated the efficacy of vaccines to prevent infections with Omicron.

To sum up, we devised and analyzed a parsimonious infectious-disease model that was able to capture the central aspects of the spread of the Omicron variant in Germany before it dominated the dynamics in early 2022, despite many uncertainties and limitations. We expect that our methodology can be used to evaluate future outbreaks, either caused by other emerging SARS-CoV-2 variants or with regard to other infectious diseases.

## Supplementary Material

bpad005_Supplementary_Data

## Data Availability

The data produced during this study has been archived on Zenodo [52].

## Code availability

The code produced during this study has been archived on Zenodo [53].
