# Supplementary material for: Modeling the impact of the Omicron infection wave in Germany
Source: Biol Methods Protoc. 2023 Mar 21;8(1):bpad005. doi: 10.1093/biomethods/bpad005 (PMC10081872; doi:10.1093/biomethods/bpad005)
Supplement: bpad005_Supplementary_Data [file bpad005_supplementary_data.zip › OUP_MAIER_OMICRON_SupplementaryMaterial.pdf]

Supplementary Material: Modeling the impact of the Omicron infection wave in Germany

1 SUPPLEMENTARY ANALYSES

1.1 Overestimation of outbreak size

The model we devised for our main analysis does not explicitly distinguish between vaccinated and unvaccinated individuals, which reduces methodical complexity and facilitates quick adaption to new data as well as the derivation of analytical results. Yet, doing so can lead to a systematic overestimation of the outbreak size up to 10%. We illustrate this overestimation by comparing two toy models that neglect waning immunity, calibration to data, or contact modulation (see Fig. S1). Note that in SIR-like models, final outbreak size is independent of any latent compartments, which we therefore omit entirely.

We first simulate (i) an SIR model with a population-averaged vaccine efficacy $v \times e$ as

$$\partial_t S = -(1 - ev)\mathcal{R}_0 \beta S I \tag{S1}$$

$$\partial_t I = (1 - ev)\mathcal{R}_0 \beta S I - \beta I \tag{S2}$$

$$\partial_t R = \beta I \tag{S3}$$

where v is the fraction of vaccinated individuals. This model is similar to Eqs. (1)–(4) in our main analysis, ignoring variants, contact modulations, and latency.

Second, we simulate (ii) an S-S_V-I-I_V-R model (subscript V marks vaccinated individuals) in which the vaccine efficacy affects only S_V individuals explicitly and breakthrough infections are counted separately, defined by

$$\partial_t S = -\mathcal{R}_0 \beta S (I + I_V) \tag{S4}$$

$$\partial_t S_V = -(1 - e)\mathcal{R}_0 \beta S_V (I + I_V) \tag{S5}$$

$$\partial_t I = \mathcal{R}_0 \beta S (I + I_V) - \beta I \tag{S6}$$

$$\partial_t I_V = (1 - e)\mathcal{R}_0 \beta S_V (I + I_V) - \beta I_V \tag{S7}$$

$$\partial_t R = \beta (I + I_V) \tag{S8}$$

Note that in model (i), the fraction of vaccinated individuals is given by the parameter v, while in model (ii), the fraction of vaccinated individuals is implemented via initial conditions $S(t = 0) = 1 - v - I_0$, $S_V(t = 0) = v$, and $I(t = 0) = I_0$.

As can be seen in Fig. S1, the first model overestimates the outbreak size by approximately 10% in the worst case (with equal parameter values for both models), in a domain of parameter values that is in line with values chosen for our main analysis.

1.2 Retrospective evaluation of the population-wide vaccine efficacy

Using Farrington's method (1), we retrospectively compute the per-calendar-week vaccine efficacy as

$$e(w) = 1 - \frac{c(w)}{1 - c(w)} \times \frac{1 - v(w - 2)}{v(w - 2)}, \tag{S9}$$

where we define as $c(w) = \Delta C_V(w)/[\Delta C_V(w) + \Delta C_I(w)]$ the share of new infections in calendar week w and as $v(w)$ the cumulative share of full vaccinations up to and including week $w - 1$ (note that in the equation above we shift $v(w)$ by two weeks to account for the period until full immunity is reached). The share v of the vaccinated population is obtained from (2) and the share of breakthrough infections (not discriminating between boostered individuals and those that were fully vaccinated but not boostered) per calendar week from the German reporting system SurvStat (3; 4). The data contains both symptomatic and asymptomatic infections. We find that the temporal evolution of vaccine efficacy as computed with Farrington's method corresponds to our "high VE", "medium reach" scenario (the latter being the assumption that about 80% of the individuals that received 2 doses in 2021 received a booster vaccination, as well), which is close to the empirically observed share of boostered individuals, see Fig. S2.

2 SUPPLEMENTARY TABLES AND FIGURES

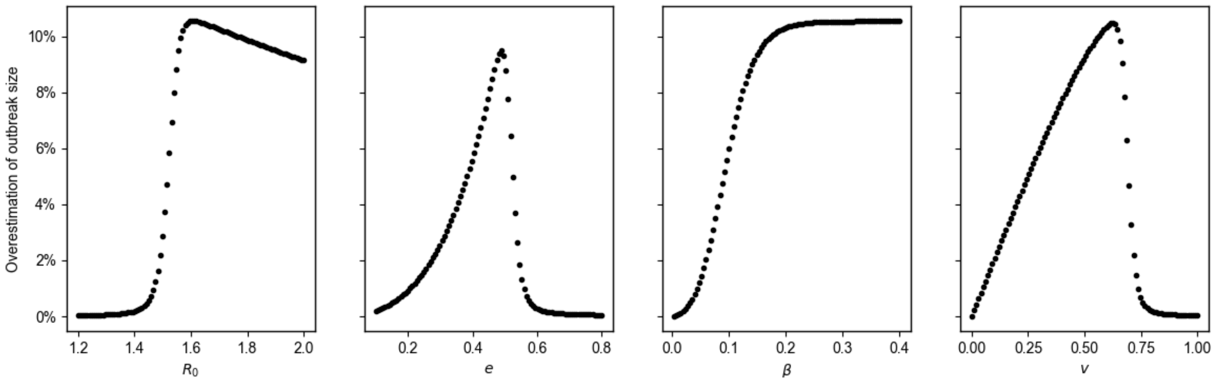

Figure S1. Overestimation of the outbreak size by employing a population-averaged vaccine SIR-model in contrast to a model that explicitly discriminates between vaccinated and unvaccinated individuals (see Eqs. (S1)-(S8)). Base parameters chosen here are vaccine efficacy against infection $e = 0.5$, recovery rate $\beta = 1/7$, reproduction ratio $\mathcal{R}_0 = 1.55$, initial fraction of vaccinated $v = 2/3$, initially infected $I_0 = 10^{-4}$. Population-averaged VE is given as $e \times v$. Overestimations are shown for different $\mathcal{R}_0$, VE e , recovery rate β, and fraction of vaccinated individuals v (from left to right).

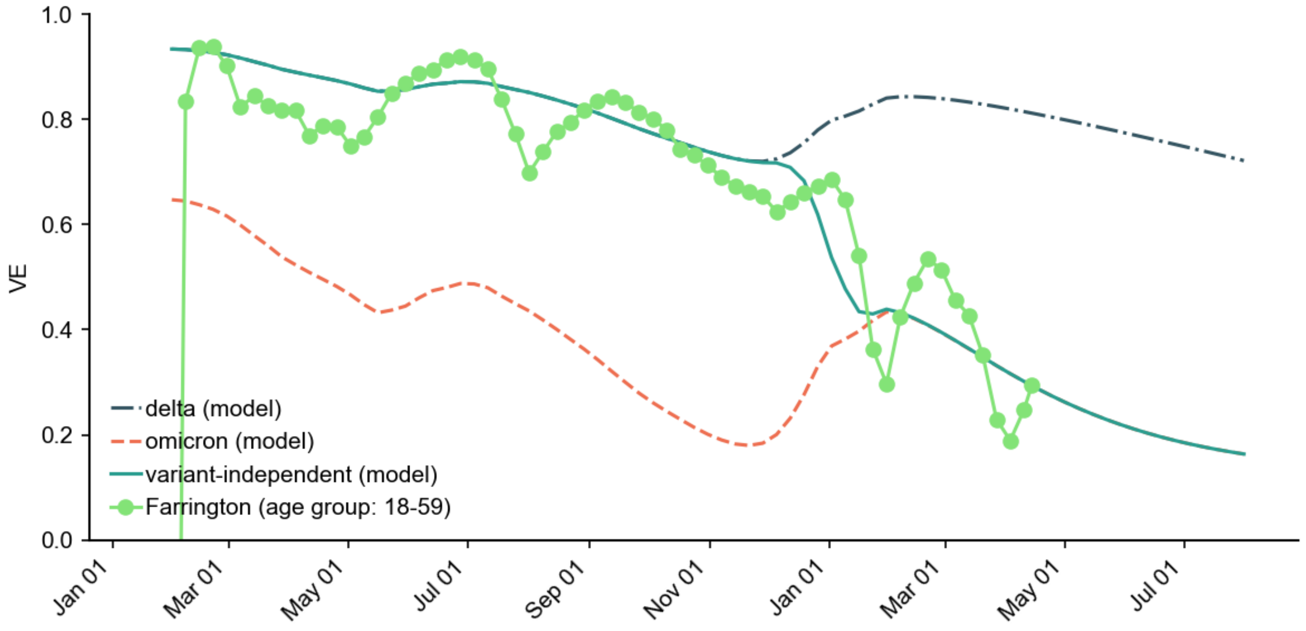

Figure S2. Retrospective comparison between vaccine efficacy obtained via Farrington's method Eq. (S9) from reported data and model scenario "high VE" and "medium reach" (cf. main text, Figs. 1–3).

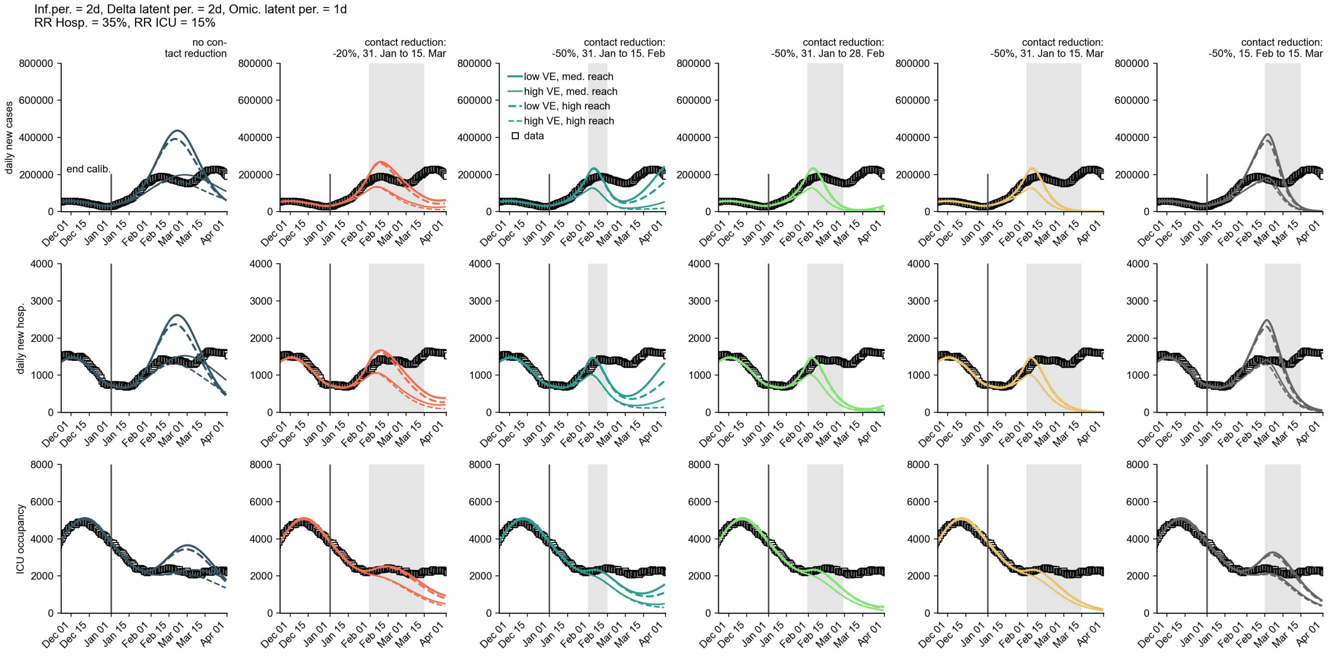

Figure S3. Different model runs for further contact reduction assumptions with a generation time of 4 days (2 days of latency + 2 days of infectious period). Intervals shaded in gray indicate the modeled periods of contact reductions.

Inf.per. [d]	Omic. Lat. [d]	Booster Reach	Booster VE	Contact Reduction	max. RR
2	1	med. reach	low VE	None	20%–25%
2	1	med. reach	low VE	-20%, Jan 31 to Mar 15	30%–35%
2	1	med. reach	low VE	-50%, Jan 31 to Feb 15	40%–45%
2	1	med. reach	low VE	-50%, Jan 31 to Feb 28	40%–45%
2	1	med. reach	low VE	-50%, Jan 31 to Mar 15	40%–45%
2	1	med. reach	low VE	-50%, Feb 15 to Mar 15	20%–25%
2	1	med. reach	high VE	None	30%–35%
2	1	med. reach	high VE	-20%, Jan 31 to Mar 15	50%–55%
2	1	med. reach	high VE	-50%, Jan 31 to Feb 15	55%–60%
2	1	med. reach	high VE	-50%, Jan 31 to Feb 28	55%–60%
2	1	med. reach	high VE	-50%, Jan 31 to Mar 15	55%–60%
2	1	med. reach	high VE	-50%, Feb 15 to Mar 15	35%–40%
2	1	high reach	low VE	None	20%–25%
2	1	high reach	low VE	-20%, Jan 31 to Mar 15	30%–35%
2	1	high reach	low VE	-50%, Jan 31 to Feb 15	40%–45%
2	1	high reach	low VE	-50%, Jan 31 to Feb 28	40%–45%
2	1	high reach	low VE	-50%, Jan 31 to Mar 15	40%–45%
2	1	high reach	low VE	-50%, Feb 15 to Mar 15	20%–25%
2	1	high reach	high VE	None	35%–40%
2	1	high reach	high VE	-20%, Jan 31 to Mar 15	50%–55%
2	1	high reach	high VE	-50%, Jan 31 to Feb 15	55%–60%
2	1	high reach	high VE	-50%, Jan 31 to Feb 28	55%–60%
2	1	high reach	high VE	-50%, Jan 31 to Mar 15	55%–60%
2	1	high reach	high VE	-50%, Feb 15 to Mar 15	40%–45%

Table S1. Maximum possible relative risk (RR) of requiring intensive care for infections with Omicron vs. infections with Delta to keep ICU occupancy below a value of 4 800 beds.

Inf.per. [d]	Omic. Lat. [d]	Booster Reach	Booster VE	Cotact Reduction	max. RR
2	2	med. reach	low VE	None	15%–20%
2	2	med. reach	low VE	-20%, Jan 31 to Mar 15	20%–25%
2	2	med. reach	low VE	-50%, Jan 31 to Feb 15	30%–35%
2	2	med. reach	low VE	-50%, Jan 31 to Feb 28	30%–35%
2	2	med. reach	low VE	-50%, Jan 31 to Mar 15	30%–35%
2	2	med. reach	low VE	-50%, Feb 15 to Mar 15	15%–20%
2	2	med. reach	high VE	None	15%–20%
2	2	med. reach	high VE	-20%, Jan 31 to Mar 15	30%–35%
2	2	med. reach	high VE	-50%, Jan 31 to Feb 15	40%–45%
2	2	med. reach	high VE	-50%, Jan 31 to Feb 28	40%–45%
2	2	med. reach	high VE	-50%, Jan 31 to Mar 15	40%–45%
2	2	med. reach	high VE	-50%, Feb 15 to Mar 15	20%–25%
2	2	high reach	low VE	None	15%–20%
2	2	high reach	low VE	-20%, Jan 31 to Mar 15	20%–25%
2	2	high reach	low VE	-50%, Jan 31 to Feb 15	30%–35%
2	2	high reach	low VE	-50%, Jan 31 to Feb 28	30%–35%
2	2	high reach	low VE	-50%, Jan 31 to Mar 15	30%–35%
2	2	high reach	low VE	-50%, Feb 15 to Mar 15	15%–20%
2	2	high reach	high VE	None	20%–25%
2	2	high reach	high VE	-20%, Jan 31 to Mar 15	30%–35%
2	2	high reach	high VE	-50%, Jan 31 to Feb 15	40%–45%
2	2	high reach	high VE	-50%, Jan 31 to Feb 28	40%–45%
2	2	high reach	high VE	-50%, Jan 31 to Mar 15	40%–45%
2	2	high reach	high VE	-50%, Feb 15 to Mar 15	20%–25%

Table S2. Maximum possible relative risk (RR) of requiring intensive care for infections with Omicron vs. infections with Delta to keep ICU occupancy below a value of 4 800 beds.

Inf.per. [d]	Omic. Lat. [d]	Booster Reach	Booster VE	Contact Reduction	max. RR
3	1	med. reach	low VE	None	15%–20%
3	1	med. reach	low VE	-20%, Jan 31 to Mar 15	25%–30%
3	1	med. reach	low VE	-50%, Jan 31 to Feb 15	35%–40%
3	1	med. reach	low VE	-50%, Jan 31 to Feb 28	35%–40%
3	1	med. reach	low VE	-50%, Jan 31 to Mar 15	35%–40%
3	1	med. reach	low VE	-50%, Feb 15 to Mar 15	15%–20%
3	1	med. reach	high VE	None	20%–25%
3	1	med. reach	high VE	-20%, Jan 31 to Mar 15	40%–45%
3	1	med. reach	high VE	-50%, Jan 31 to Feb 15	45%–50%
3	1	med. reach	high VE	-50%, Jan 31 to Feb 28	45%–50%
3	1	med. reach	high VE	-50%, Jan 31 to Mar 15	45%–50%
3	1	med. reach	high VE	-50%, Feb 15 to Mar 15	25%–30%
3	1	high reach	low VE	None	15%–20%
3	1	high reach	low VE	-20%, Jan 31 to Mar 15	25%–30%
3	1	high reach	low VE	-50%, Jan 31 to Feb 15	35%–40%
3	1	high reach	low VE	-50%, Jan 31 to Feb 28	35%–40%
3	1	high reach	low VE	-50%, Jan 31 to Mar 15	35%–40%
3	1	high reach	low VE	-50%, Feb 15 to Mar 15	20%–25%
3	1	high reach	high VE	None	25%–30%
3	1	high reach	high VE	-20%, Jan 31 to Mar 15	40%–45%
3	1	high reach	high VE	-50%, Jan 31 to Feb 15	45%–50%
3	1	high reach	high VE	-50%, Jan 31 to Feb 28	45%–50%
3	1	high reach	high VE	-50%, Jan 31 to Mar 15	45%–50%
3	1	high reach	high VE	-50%, Feb 15 to Mar 15	30%–35%

Table S3. Maximum possible relative risk (RR) of requiring intensive care for infections with Omicron vs. infections with Delta to keep ICU occupancy below a value of 4 800 beds.

Inf.per. [d]	Omic. Lat. [d]	Booster Reach	Booster VE	Contact Reduction	max. RR
3	2	med. reach	low VE	None	10%–15%
3	2	med. reach	low VE	-20%, Jan 31 to Mar 15	15%–20%
3	2	med. reach	low VE	-50%, Jan 31 to Feb 15	20%–25%
3	2	med. reach	low VE	-50%, Jan 31 to Feb 28	25%–30%
3	2	med. reach	low VE	-50%, Jan 31 to Mar 15	25%–30%
3	2	med. reach	low VE	-50%, Feb 15 to Mar 15	15%–20%
3	2	med. reach	high VE	None	15%–20%
3	2	med. reach	high VE	-20%, Jan 31 to Mar 15	25%–30%
3	2	med. reach	high VE	-50%, Jan 31 to Feb 15	35%–40%
3	2	med. reach	high VE	-50%, Jan 31 to Feb 28	35%–40%
3	2	med. reach	high VE	-50%, Jan 31 to Mar 15	35%–40%
3	2	med. reach	high VE	-50%, Feb 15 to Mar 15	15%–20%
3	2	high reach	low VE	None	10%–15%
3	2	high reach	low VE	-20%, Jan 31 to Mar 15	15%–20%
3	2	high reach	low VE	-50%, Jan 31 to Feb 15	25%–30%
3	2	high reach	low VE	-50%, Jan 31 to Feb 28	25%–30%
3	2	high reach	low VE	-50%, Jan 31 to Mar 15	25%–30%
3	2	high reach	low VE	-50%, Feb 15 to Mar 15	15%–20%
3	2	high reach	high VE	None	15%–20%
3	2	high reach	high VE	-20%, Jan 31 to Mar 15	25%–30%
3	2	high reach	high VE	-50%, Jan 31 to Feb 15	35%–40%
3	2	high reach	high VE	-50%, Jan 31 to Feb 28	35%–40%
3	2	high reach	high VE	-50%, Jan 31 to Mar 15	35%–40%
3	2	high reach	high VE	-50%, Feb 15 to Mar 15	20%–25%

Table S4. Maximum possible relative risk (RR) of requiring intensive care for infections with Omicron vs. infections with Delta to keep ICU occupancy below a value of 4 800 beds.

Inf.per. [d]	Omic. Lat. [d]	Booster Reach	Booster VE	Contact Reduction	Om. Cases Total [Mil]
2	1	med. reach	low VE	None	18.9
2	1	med. reach	low VE	-20%, Jan 31 to Mar 15	11.5
2	1	med. reach	low VE	-50%, Jan 31 to Feb 15	10.2
2	1	med. reach	low VE	-50%, Jan 31 to Feb 28	6.6
2	1	med. reach	low VE	-50%, Jan 31 to Mar 15	6.3
2	1	med. reach	low VE	-50%, Feb 15 to Mar 15	12.9
2	1	med. reach	high VE	None	11.5
2	1	med. reach	high VE	-20%, Jan 31 to Mar 15	5.7
2	1	med. reach	high VE	-50%, Jan 31 to Feb 15	4.6
2	1	med. reach	high VE	-50%, Jan 31 to Feb 28	3.8
2	1	med. reach	high VE	-50%, Jan 31 to Mar 15	3.7
2	1	med. reach	high VE	-50%, Feb 15 to Mar 15	6.6
2	1	high reach	low VE	None	17.4
2	1	high reach	low VE	-20%, Jan 31 to Mar 15	10.5
2	1	high reach	low VE	-50%, Jan 31 to Feb 15	8.8
2	1	high reach	low VE	-50%, Jan 31 to Feb 28	6.4
2	1	high reach	low VE	-50%, Jan 31 to Mar 15	6.2
2	1	high reach	low VE	-50%, Feb 15 to Mar 15	12.1
2	1	high reach	high VE	None	9.4
2	1	high reach	high VE	-20%, Jan 31 to Mar 15	5.2
2	1	high reach	high VE	-50%, Jan 31 to Feb 15	4.1
2	1	high reach	high VE	-50%, Jan 31 to Feb 28	3.7
2	1	high reach	high VE	-50%, Jan 31 to Mar 15	3.7
2	1	high reach	high VE	-50%, Feb 15 to Mar 15	6.2

Table S5. Outbreak sizes expected according to model by April 1, 2022 (cumulative reported cases of Omicron infections assuming constant unreported cases).

Inf.per. [d]	Omic. Lat. [d]	Booster Reach	Booster VE	Contact Reduction	Om. Cases Total [Mil]
2	2	med. reach	low VE	None	23.6
2	2	med. reach	low VE	-20%, Jan 31 to Mar 15	16.9
2	2	med. reach	low VE	-50%, Jan 31 to Feb 15	16.0
2	2	med. reach	low VE	-50%, Jan 31 to Feb 28	10.1
2	2	med. reach	low VE	-50%, Jan 31 to Mar 15	8.9
2	2	med. reach	low VE	-50%, Feb 15 to Mar 15	17.5
2	2	med. reach	high VE	None	17.5
2	2	med. reach	high VE	-20%, Jan 31 to Mar 15	10.4
2	2	med. reach	high VE	-50%, Jan 31 to Feb 15	8.8
2	2	med. reach	high VE	-50%, Jan 31 to Feb 28	5.9
2	2	med. reach	high VE	-50%, Jan 31 to Mar 15	5.5
2	2	med. reach	high VE	-50%, Feb 15 to Mar 15	10.7
2	2	high reach	low VE	None	22.4
2	2	high reach	low VE	-20%, Jan 31 to Mar 15	15.7
2	2	high reach	low VE	-50%, Jan 31 to Feb 15	14.3
2	2	high reach	low VE	-50%, Jan 31 to Feb 28	9.5
2	2	high reach	low VE	-50%, Jan 31 to Mar 15	8.7
2	2	high reach	low VE	-50%, Feb 15 to Mar 15	16.6
2	2	high reach	high VE	None	15.4
2	2	high reach	high VE	-20%, Jan 31 to Mar 15	9.2
2	2	high reach	high VE	-50%, Jan 31 to Feb 15	7.4
2	2	high reach	high VE	-50%, Jan 31 to Feb 28	5.6
2	2	high reach	high VE	-50%, Jan 31 to Mar 15	5.4
2	2	high reach	high VE	-50%, Feb 15 to Mar 15	10.0

Table S6. Outbreak sizes expected according to model by April 1, 2022 (cumulative reported cases of Omicron infections assuming constant unreported cases).

Inf.per. [d]	Omic. Lat. [d]	Booster Reach	Booster VE	Contact Reduction	Om. Cases Total [Mil]
3	1	med. reach	low VE	None	22.6
3	1	med. reach	low VE	-20%, Jan 31 to Mar 15	15.2
3	1	med. reach	low VE	-50%, Jan 31 to Feb 15	14.9
3	1	med. reach	low VE	-50%, Jan 31 to Feb 28	8.7
3	1	med. reach	low VE	-50%, Jan 31 to Mar 15	7.5
3	1	med. reach	low VE	-50%, Feb 15 to Mar 15	15.4
3	1	med. reach	high VE	None	15.8
3	1	med. reach	high VE	-20%, Jan 31 to Mar 15	8.6
3	1	med. reach	high VE	-50%, Jan 31 to Feb 15	7.5
3	1	med. reach	high VE	-50%, Jan 31 to Feb 28	4.9
3	1	med. reach	high VE	-50%, Jan 31 to Mar 15	4.6
3	1	med. reach	high VE	-50%, Feb 15 to Mar 15	8.9
3	1	high reach	low VE	None	21.2
3	1	high reach	low VE	-20%, Jan 31 to Mar 15	14.0
3	1	high reach	low VE	-50%, Jan 31 to Feb 15	13.1
3	1	high reach	low VE	-50%, Jan 31 to Feb 28	8.1
3	1	high reach	low VE	-50%, Jan 31 to Mar 15	7.3
3	1	high reach	low VE	-50%, Feb 15 to Mar 15	14.5
3	1	high reach	high VE	None	13.5
3	1	high reach	high VE	-20%, Jan 31 to Mar 15	7.6
3	1	high reach	high VE	-50%, Jan 31 to Feb 15	6.2
3	1	high reach	high VE	-50%, Jan 31 to Feb 28	4.7
3	1	high reach	high VE	-50%, Jan 31 to Mar 15	4.5
3	1	high reach	high VE	-50%, Feb 15 to Mar 15	8.2

Table S7. Outbreak sizes expected according to model by April 1, 2022 (cumulative reported cases of Omicron infections assuming constant unreported cases).

Inf.per. [d]	Omic. Lat. [d]	Booster Reach	Booster VE	Contact Reduction	Om. Cases Total [Mil]
3	2	med. reach	low VE	None	26.5
3	2	med. reach	low VE	-20%, Jan 31 to Mar 15	20.3
3	2	med. reach	low VE	-50%, Jan 31 to Feb 15	20.1
3	2	med. reach	low VE	-50%, Jan 31 to Feb 28	13.0
3	2	med. reach	low VE	-50%, Jan 31 to Mar 15	10.6
3	2	med. reach	low VE	-50%, Feb 15 to Mar 15	19.8
3	2	med. reach	high VE	None	21.1
3	2	med. reach	high VE	-20%, Jan 31 to Mar 15	13.8
3	2	med. reach	high VE	-50%, Jan 31 to Feb 15	12.7
3	2	med. reach	high VE	-50%, Jan 31 to Feb 28	7.9
3	2	med. reach	high VE	-50%, Jan 31 to Mar 15	6.8
3	2	med. reach	high VE	-50%, Feb 15 to Mar 15	13.2
3	2	high reach	low VE	None	25.4
3	2	high reach	low VE	-20%, Jan 31 to Mar 15	19.1
3	2	high reach	low VE	-50%, Jan 31 to Feb 15	18.4
3	2	high reach	low VE	-50%, Jan 31 to Feb 28	12.1
3	2	high reach	low VE	-50%, Jan 31 to Mar 15	10.2
3	2	high reach	low VE	-50%, Feb 15 to Mar 15	18.9
3	2	high reach	high VE	None	19.0
3	2	high reach	high VE	-20%, Jan 31 to Mar 15	12.3
3	2	high reach	high VE	-50%, Jan 31 to Feb 15	10.7
3	2	high reach	high VE	-50%, Jan 31 to Feb 28	7.2
3	2	high reach	high VE	-50%, Jan 31 to Mar 15	6.5
3	2	high reach	high VE	-50%, Feb 15 to Mar 15	12.2

Table S8. Outbreak sizes expected according to model by April 1, 2022 (cumulative reported cases of Omicron infections assuming constant unreported cases).

β^{-1} [d]	ω_o^{-1} [d]	Reach	Booster VE	Cont.red.	$\max(J_C)/10^3$	$\max(J_H)/10^3$	$\max(U)/10^3$
2	1	med.	low VE	None	437	2.6	3.7
2	1	med.	low VE	-20%, 01.31.–03.15.	268	1.7	2.5
2	1	med.	low VE	-50%, 01.31.–02.15.	233	1.5	2.3
2	1	med.	low VE	-50%, 01.31.–02.28.	233	1.5	2.3
2	1	med.	low VE	-50%, 01.31.–03.15.	233	1.5	2.3
2	1	med.	low VE	-50%, 02.15.–03.15.	416	2.5	3.3
2	1	med.	high VE	None	198	1.5	2.3
2	1	med.	high VE	-20%, 01.31.–03.15.	135	1.1	–
2	1	med.	high VE	-50%, 01.31.–02.15.	127	1.0	–
2	1	med.	high VE	-50%, 01.31.–02.28.	127	1.0	–
2	1	med.	high VE	-50%, 01.31.–03.15.	127	1.0	–
2	1	med.	high VE	-50%, 02.15.–03.15.	183	1.4	2.1
2	1	high	low VE	None	392	2.4	3.4
2	1	high	low VE	-20%, 01.31.–03.15.	260	1.6	2.5
2	1	high	low VE	-50%, 01.31.–02.15.	231	1.5	2.3
2	1	high	low VE	-50%, 01.31.–02.28.	231	1.5	2.3
2	1	high	low VE	-50%, 01.31.–03.15.	231	1.5	2.3
2	1	high	low VE	-50%, 02.15.–03.15.	382	2.3	3.1
2	1	high	high VE	None	166	1.3	2.1
2	1	high	high VE	-20%, 01.31.–03.15.	132	1.1	–
2	1	high	high VE	-50%, 01.31.–02.15.	126	1.0	–
2	1	high	high VE	-50%, 01.31.–02.28.	126	1.0	–
2	1	high	high VE	-50%, 01.31.–03.15.	126	1.0	–
2	1	high	high VE	-50%, 02.15.–03.15.	165	1.3	2.1

Table S9. Maximum values of all local maxima of incidence, hospitalization incidence and ITS occupancy in the period January 2022 to April 2022. Curves with entries "–" show no local maxima. Here, RR Hosp. = 0.35 and RR ICU = 0.15 were assumed.

β^{-1} [d]	ω_o^{-1} [d]	Reach	Booster VE	Cont.red.	$\max(J_C)/10^3$	$\max(J_H)/10^3$	$\max(U)/10^3$
2	2	med.	low VE	None	598	3.6	4.8
2	2	med.	low VE	-20%, 01.31.–03.15.	384	2.4	3.3
2	2	med.	low VE	-50%, 01.31.–02.15.	310	1.9	2.7
2	2	med.	low VE	-50%, 01.31.–02.28.	310	1.9	2.7
2	2	med.	low VE	-50%, 01.31.–03.15.	310	1.9	2.7
2	2	med.	low VE	-50%, 02.15.–03.15.	579	3.4	4.3
2	2	med.	high VE	None	355	2.7	3.8
2	2	med.	high VE	-20%, 01.31.–03.15.	213	1.6	2.5
2	2	med.	high VE	-50%, 01.31.–02.15.	187	1.4	2.3
2	2	med.	high VE	-50%, 01.31.–02.28.	187	1.4	2.3
2	2	med.	high VE	-50%, 01.31.–03.15.	187	1.4	2.3
2	2	med.	high VE	-50%, 02.15.–03.15.	321	2.4	3.2
2	2	high	low VE	None	554	3.3	4.6
2	2	high	low VE	-20%, 01.31.–03.15.	370	2.3	3.3
2	2	high	low VE	-50%, 01.31.–02.15.	307	1.9	2.7
2	2	high	low VE	-50%, 01.31.–02.28.	307	1.9	2.7
2	2	high	low VE	-50%, 01.31.–03.15.	307	1.9	2.7
2	2	high	low VE	-50%, 02.15.–03.15.	543	3.2	4.2
2	2	high	high VE	None	307	2.4	3.5
2	2	high	high VE	-20%, 01.31.–03.15.	207	1.6	2.5
2	2	high	high VE	-50%, 01.31.–02.15.	185	1.4	2.3
2	2	high	high VE	-50%, 01.31.–02.28.	185	1.4	2.3
2	2	high	high VE	-50%, 01.31.–03.15.	185	1.4	2.3
2	2	high	high VE	-50%, 02.15.–03.15.	294	2.2	3.1

Table S10. Maximum values of all local maxima of incidence, hospitalization incidence and ITS occupancy in the period January 2022 to April 2022. Curves with entries "−" show no local maxima. Here, RR Hosp. = 0.35 and RR ICU = 0.15 were assumed.

β^{-1} [d]	ω_o^{-1} [d]	Reach	Booster VE	Cont.red.	$\max(J_C)/10^3$	$\max(J_H)/10^3$	$\max(U)/10^3$
3	1	med.	low VE	None	539	3.2	4.4
3	1	med.	low VE	-20%, 01.31.–03.15.	312	1.9	2.8
3	1	med.	low VE	-50%, 01.31.–02.15.	343	1.9	2.4
3	1	med.	low VE	-50%, 01.31.–02.28.	252	1.6	2.4
3	1	med.	low VE	-50%, 01.31.–03.15.	252	1.6	2.4
3	1	med.	low VE	-50%, 02.15.–03.15.	498	2.9	3.7
3	1	med.	high VE	None	302	2.3	3.3
3	1	med.	high VE	-20%, 01.31.–03.15.	166	1.3	2.2
3	1	med.	high VE	-50%, 01.31.–02.15.	150	1.2	–
3	1	med.	high VE	-50%, 01.31.–02.28.	150	1.2	–
3	1	med.	high VE	-50%, 01.31.–03.15.	150	1.2	–
3	1	med.	high VE	-50%, 02.15.–03.15.	254	1.9	2.6
3	1	high	low VE	None	490	2.9	4.2
3	1	high	low VE	-20%, 01.31.–03.15.	299	1.9	2.8
3	1	high	low VE	-50%, 01.31.–02.15.	249	1.6	2.4
3	1	high	low VE	-50%, 01.31.–02.28.	249	1.6	2.4
3	1	high	low VE	-50%, 01.31.–03.15.	249	1.6	2.4
3	1	high	low VE	-50%, 02.15.–03.15.	462	2.7	3.6
3	1	high	high VE	None	249	1.9	2.9
3	1	high	high VE	-20%, 01.31.–03.15.	162	1.3	–
3	1	high	high VE	-50%, 01.31.–02.15.	148	1.2	–
3	1	high	high VE	-50%, 01.31.–02.28.	148	1.2	–
3	1	high	high VE	-50%, 01.31.–03.15.	148	1.2	–
3	1	high	high VE	-50%, 02.15.–03.15.	231	1.8	2.5

Table S11. Maximum values of all local maxima of incidence, hospitalization incidence and ITS occupancy in the period January 2022 to April 2022. Curves with entries "−" show no local maxima. Here, RR Hosp. = 0.35 and RR ICU = 0.15 were assumed.

β^{-1} [d]	ω_o^{-1} [d]	Reach	Booster VE	Cont.red.	$\max(J_C)/10^3$	$\max(J_H)/10^3$	$\max(U)/10^3$
3	2	med.	low VE	None	680	4.0	5.3
3	2	med.	low VE	-20%, 01.31.–03.15.	438	2.7	3.8
3	2	med.	low VE	-50%, 01.31.–02.15.	385	2.2	3.1
3	2	med.	low VE	-50%, 01.31.–02.28.	325	2.0	2.8
3	2	med.	low VE	-50%, 01.31.–03.15.	325	2.0	2.8
3	2	med.	low VE	-50%, 02.15.–03.15.	647	3.8	4.7
3	2	med.	high VE	None	450	3.4	4.7
3	2	med.	high VE	-20%, 01.31.–03.15.	254	2.0	2.9
3	2	med.	high VE	-50%, 01.31.–02.15.	208	1.6	2.4
3	2	med.	high VE	-50%, 01.31.–02.28.	208	1.6	2.4
3	2	med.	high VE	-50%, 01.31.–03.15.	208	1.6	2.4
3	2	med.	high VE	-50%, 02.15.–03.15.	391	2.9	3.7
3	2	high	low VE	None	635	3.8	5.2
3	2	high	low VE	-20%, 01.31.–03.15.	416	2.6	3.7
3	2	high	low VE	-50%, 01.31.–02.15.	332	2.0	2.9
3	2	high	low VE	-50%, 01.31.–02.28.	321	2.0	2.8
3	2	high	low VE	-50%, 01.31.–03.15.	321	2.0	2.8
3	2	high	low VE	-50%, 02.15.–03.15.	610	3.6	4.6
3	2	high	high VE	None	394	3.0	4.4
3	2	high	high VE	-20%, 01.31.–03.15.	243	1.9	2.8
3	2	high	high VE	-50%, 01.31.–02.15.	206	1.6	2.4
3	2	high	high VE	-50%, 01.31.–02.28.	206	1.6	2.4
3	2	high	high VE	-50%, 01.31.–03.15.	206	1.6	2.4
3	2	high	high VE	-50%, 02.15.–03.15.	360	2.7	3.6

Table S12. Maximum values of all local maxima of incidence, hospitalization incidence and ITS occupancy in the period January 2022 to April 2022. Curves with entries "−" show no local maxima. Here, RR Hosp. = 0.35 and RR ICU = 0.15 were assumed.

REFERENCES

[1]Farrington CP. Estimation of Vaccine Effectiveness Using the Screening Method. International Journal of Epidemiology. 1993;22(4):742-6. Available from: https://academic.oup.com/ije/article-lookup/doi/10.1093/ije/22.4.742.

[2]Robert Koch Institute. COVID-19-Impfungen in Deutschland. Zenodo; 2021. Type: dataset. Available from: https://zenodo.org/record/5126652.

[3]Faensen, D and Krause, G. SurvStat@RKI – a web-based solution to query surveillance data in Germany. Weekly releases (1997–2007) Euro Surveill. 2004;8(22). Available from: https://www.eurosurveillance.org/content/10.2807/esw.08.22.02477-en.

[4]Robert Koch Institute. SurvStat@RKI 2.0 - Web-basierte Abfrage der Meldedaten gemäß Infektionsschutzgesetz (IfSG); 2021.
